# Supplementary material for: CopySwitch—in vivo Optimization of Gene Copy Numbers for Heterologous Gene Expression in Bacillus subtilis
Source: Front Bioeng Biotechnol. 2019 Jan 8;6:207. doi: 10.3389/fbioe.2018.00207 (PMC6331482; doi:10.3389/fbioe.2018.00207)
Supplement: Supplementary file 1 [file Data_Sheet_1.pdf]

Supplementary Material

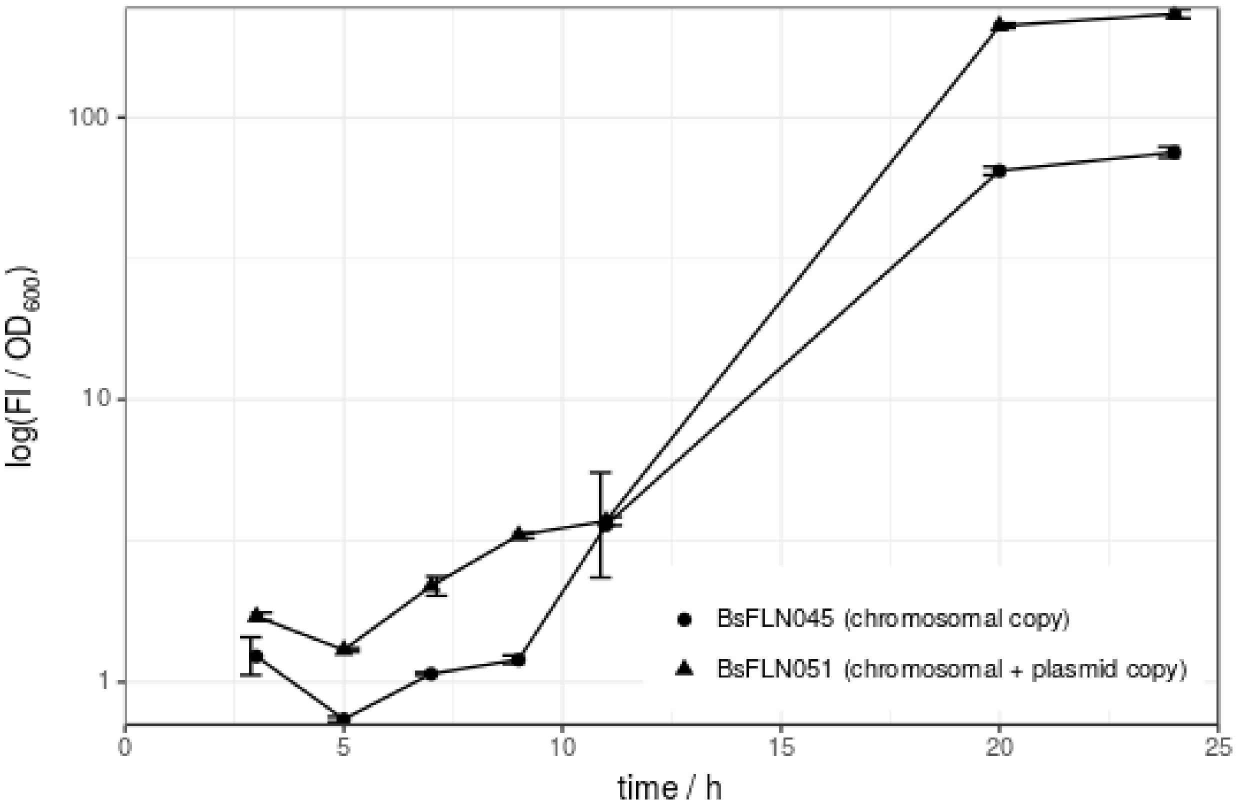

Figure S1: Comparison of fluorescence intensity normalized to OD_{600} of cell populations containing only a chromosomal copy (BsFLN045) or a chromosomal plus a plasmid copy (BsFLN051) of P_{acoA}-*GFPmut2*. Measurements were carried out in a PHERAstar FSX spectrophotometer (BMG Labtech, Ortenberg, Germany). Replicate number n = 4 with error bars showing standard deviation.

Plasmid sequence list (FASTA format and annotated maps)

>p14035_plcBReT-sh

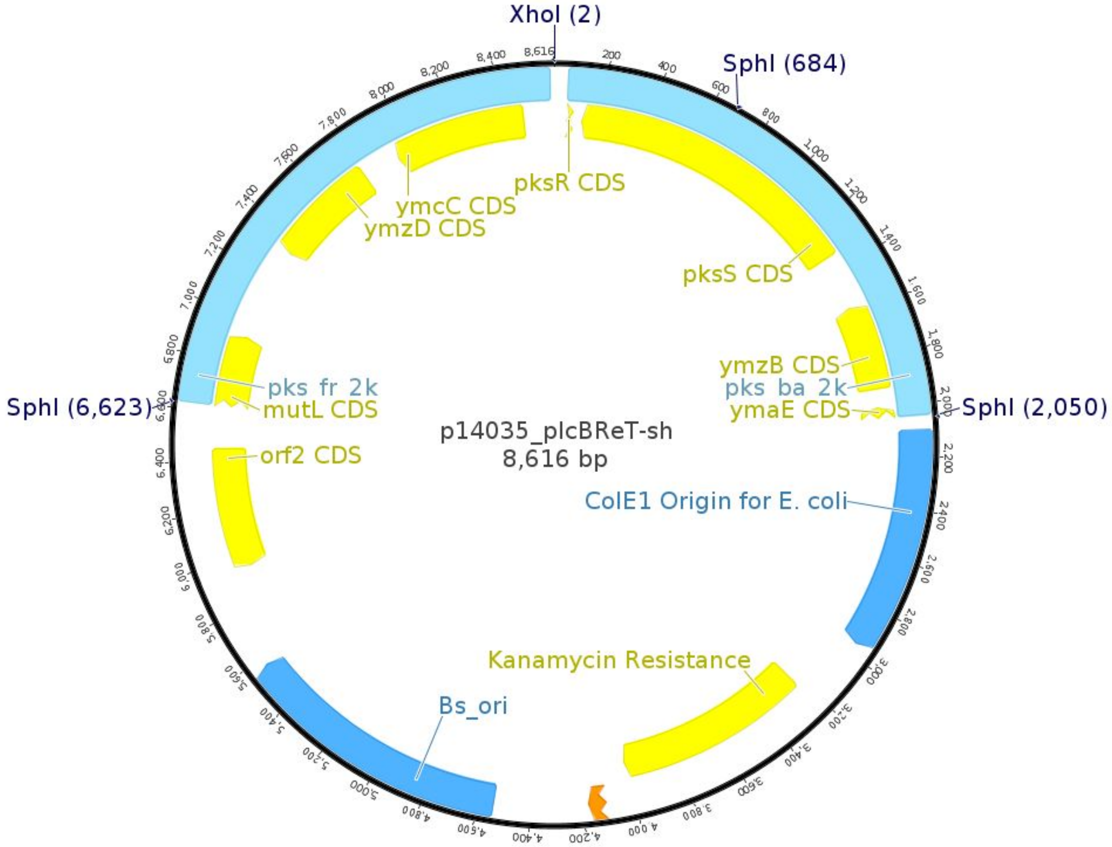

CTCGAGGCCTACCGTTCGTATAGCATACATTATACGAACGGTAGGCCTCTAGATGAATTGG
TGAAGCGCTGAGAAAACACAAACGCCCCCTCTTTTAAAAGGGGGCGTTTTGAATGTTATTTT
GAAAGTGAAACAGGGAGACTTTCTAATCCTCTTAAAAAGACATTTTTTCTCCATTGAATGTC
ATCAGGTGCAACCGCAAGTTCAATATCAGGAAATCTCTTCAAAAGTGCTTTAAATGCAATGT
GGCCTTCCAGCCTGGCAAGAGGCGCTCCTAAGCAGAAATGAATGCCAAAACCAAAAGAAA
TATGTCTATTAGGCGACCGATTTATATTTAATATTTCGGGGTTCTCAAAAAAATTCGGGTCG
CGATTGGCAGATCCGATGCCTATAAAAATCATGTCTCCTCTTTTGATCGAATGCCCCTTATA
TGTAAAGTCTTCGATGGCCCACCGATTTGCCATCATAACGACAGGTGAGGTGTATCGCAGC
AATTCTTCAACCGCTGTAGCGATCATTTCAGGCTGCTGCTTGAGCTTCTCACATTCCTTCTT
GTGCTGCAGCAATGCGAGGGTGCCTGAGCCGAGTAAGTTAACAGTTGTTTCAAGGCCGGC
TACAACGAGCAAGAACAGCATCGAATAGAGCTCTTTTTCGCTTAACTTGCTGCCGTTTTCCT
CAGCATGCACAAGTTTGCTGATTAAATCGTCTTTTGGCTTTATTCTTCTGTCATGGATCAGC
TTAGCGATATAATCTTTAAATTCACGAAGGGCCTGATTTGTCAGCTCTCTATTACCTTCAGA
GGTATCAACCATCGCATTGGTCCAGATTTGAAACTGTGACCGATCTTCTTTTGGGATTCCCA
TCAATTCAGATATAACAATAAAAGGCAAAGGGGAAGCGAAGGATTTCATGATATCCGCTTTA
TTTTCTTTTTCCATTTCATCTAAAAGCTGTTCAGCAATTTGTTCAATGCTGCCGCGCAGATTT
TCAATGGTTCGGGGAGTAAATGCTTGATGAACAAGTGATCTCAGGCGGGTATGGTCAGGT
GTGTCTTTTGCCAGCATATGATCGGATACAAAATCGATATCTTCACTAACGTTGAGCATTTT

GATTTGTTCTTGGTTCATCACATTTTTTACGTCTCTTGTAATTCGATTGTCTTTTAAAAAGGC
CATACAATCATCGTATCGGGTAATTAACCAGGCCGGATATGTGGCTCCGAACCGTTTTAATT
CAAATCGGTGAATGGGCTCTTCCTCTCTAAATCGTCCTAAAACTGAAAAAGGATTGTGATGA
AACTCTTTACCATGCGGATGAAACATCAATTTTTCCATTTGCATTCTCCTCGCCTAATAGGG
TAAATAGATGAATCAAATTGCTGAATTAGTTTACAAAAAACAGAATGATTTGAAATGTAATCC
TGTCTCTAAAACTATATATCTATCTTAGGCGTCATTCAATAGGGAGAAGAACGAAAAAAGTG
AAAAAACGGCTCGATATAAAGCAGCGCCTTTGAACGAAAGCTCAAAGGCGCTACGCTGTAT
TATTTTGATGAAAGTGGCTGTCAGCTGTGCTGGATATCAATTGTATATACTGCACGATCTGT
TACGACCTTCAATCCTTCGTTTTCTTGGTGAATATGAAGCTCACCTAATAAAGGAATCTCAT
AGGCATTTTGCGGAAGCGGTACGTTTCCGTCCTCAGTGAATGTTGTTCCTTCGCCTTCTAA
AACAAGCTCTTCTTTCGCAACATAATCATCAGGATGATTCGTACTGCGTATAGCCACTTTTT
GAAGGTGGAGAACAATCTGATCCAAGTCTGATATCTCTTCATCATGGGTCAATTCACCTTTT
CTAATATCAAGCGTCTGGCCGACTTTTGATTCCAGCCATTGAGAAAGCTGTGTATTCGTGT
GTGCCATCAATATTCCTCCTTTTGATCTACACGATACTATTCCCAATTGCTGCATCTTTTACA
CGGGAAAGAGCCGCCCCACTCTTTATGTATGAAACAGTTCGCTGTTGAGTGCCTTGATATA
GCGTACGCATGCACGCGTTGATCAGGCGCCTCGTTCCACTGAGCGTCAGACCCCGTAGAA
AAGATCAAAGGATCTTCTTGAGATCCTTTTTTTCTGCGCGTAATCTGCTGCTTGCAAACAAA
AAAACCACCGCTACCAGCGGTGGTTTGTTTGCCGGATCAAGAGCTACCAACTCTTTTTCCG
AAGGTAACTGGCTTCAGCAGAGCGCAGATACCAAATACTGTTCTTCTAGTGTAGCCGTAGT
TAGGCCACCACTTCAAGAACTCTGTAGCACCGCCTACATACCTCGCTCTGCTAATCCTGTT
ACCAGTGGCTGCTGCCAGTGGCGATAAGTCGTGTCTTACCGGGTTGGACTCAAGACGATA
GTTACCGGATAAGGCGCAGCGGTCGGGCTGAACGGGGGGTTCGTGCACACAGCCCAGCT
TGGAGCGAACGACCTACACCGAACTGAGATACCTACAGCGTGAGCTATGAGAAAGCGCCA
CGCTTCCCGAAGGGAGAAAGGCGGACAGGTATCCGGTAAGCGGCAGGGTCGGAACAGGA
GAGCGCACGAGGGAGCTTCCAGGGGGAAACGCCTGGTATCTTTATAGTCCTGTCGGGTTT
CGCCACCTCTGACTTGAGCGTCGATTTTTGTGATGCTCGTCAGGGGGGCGGAGCCTATGG
AAAAACGCCAGCAACGCGGCCTTTTTACGGTTCCTGGCCTTTTGCTGGCCTTTTGCTCACA
TGTTCTTTCCTGCGTTATCCCCTGATTCTGTGGATAACCGTATTACCGCCTTTGAGTGAGCT
GATACCGCTCGCCGCAGCCGAACGACCGAGCGCAGCGAGTCAGTGAGCGAGGAAGCGGA
AGAGCGCCCAATACGCAAACCGCCTCTCCCCGCGCGTTGGCCGATTCATTAATGCAGGTT
GATCACCTAGAGGATCCCCCAAAGCATAAAAACTTGCATGGACTAATGCTTGAAACCCAGG
ACAATAACCTTATAGCTTGTAAATTCTATCATAATTGTGGTTTCAAAATCGGCTCCGTCGATA
CTATGTTATACGCCAACTTTGAAAACAACTTTGAAAAAGCTGTTTTCTGGTATTTAAGGTTTT
AGAATGCAAGGAACAGTGAATTGGAGTTCGTCTTGTTATAATTAGCTTCTTGGGGTATCTTT
AAATACTGTAGAAAAGAGGAAGGAAATAATAAATGGCTAAAATGAGAATATCACCGGAATTG
AAAAAACTGATCGAAAAATACCGCTGCGTAAAAGATACGGAAGGAATGTCTCCTGCTAAGG
TATATAAGCTGGTGGGAGAAAATGAAAACCTATATTTAAAAATGACGGACAGCCGGTATAAA
GGGACCACCTATGATGTGGAACGGGAAAAGGACATGATGCTATGGCTGGAAGGAAAGCTG
CCTGTTCCAAAGGTCCTGCACTTTGAACGGCATGATGGCTGGAGCAATCTGCTCATGAGTG
AGGCCGATGGCGTCCTTTGCTCGGAAGAGTATGAAGATGAACAAAGCCCTGAAAAGATTAT
CGAGCTGTATGCGGAGTGCATCAGGCTCTTTCACTCCATCGACATATCGGATTGTCCCTAT
ACGAATAGCTTAGACAGCCGCTTAGCCGAATTGGATTACTTACTGAATAACGATCTGGCCG
ATGTGGATTGCGAAAACTGGGAAGAAGACACTCCATTTAAAGATCCGCGCGAGCTGTATGA
TTTTTTAAAGACGGAAAAGCCCGAAGAGGAACTTGTCTTTTCCCACGGCGACCTGGGAGAC
AGCAACATCTTTGTGAAAGATGGCAAAGTAAGTGGCTTTATTGATCTTGGGAGAAGCGGCA
GGGCGGACAAGTGGTATGACATTGCCTTCTGCGTCCGGTCGATCAGGGAGGATATCGGG
GAAGAACAGTATGTCGAGCTATTTTTTGACTTACTGGGGATCAAGCCTGATTGGGAGAAAA
TAAAATATTATATTTTACTGGATGAATTGTTTTAGTACCTAGATTTAGATGTCTAAAAAGCCT

GCAGTCGCGAGGCCTCGACGGATCCCCAAAGCACTTGCATAGGCTAATGCCTGGCTTGGT
TTGTTGAGACATTACCTGAGAGCCAAAAAACTTTTTATGAATATGAATTAAAAAAAAGAACCA
ACAAAGGCTGAGACAGACTCCAAACGAGTCTGTTTTTTTAAAAAAAATATTAGGAGCATTGA
ATATATATTAGAGAATTAAGAAAGACATGGGAATAAAAATATTTTAAATCCAGTAAAAATATG
ATAAGATTATTTCAGAATATGAAGAACTCTGTTTGTTTTTGATGAAAAAACAAACAAAAAAAA
TCCACCTAACGGAATCTCAATTTAACTAACAGCGGCCAAACTGAGAAGTTAAATTTGAGAAG
GGGAAAAGGCGGATTTATACTTGTATTTAACTATCTCCATTTTAACATTTTATTAAACCCCAT
ACAAGTGAAAATCCTCTTTTACACTGTTCCTTTAGGTGATCGCGGAGGGACATTATGAGTGA
AGTAAACCTAAAAGGAAATACAGATGAATTAGTGTATTATCGACAGCAAACCACTGGAAATA
AAATCGCCAGGAAGAGAATCAAAAAAGGGAAAGAAGAAGTTTATTATGTTGCTGAAACGGA
AGAGAAGATATGGACAGAAGAGCAAATAAAAAACTTTTCTTTAGACAAATTTGGTACGCATA
TACCTTACATAGAAGGTCATTATACAATCTTAAATAATTACTTCTTTGATTTTTGGGGCTATTT
TTTAGGTGCTGAAGGAATTGCGCTCTATGCTCACCTAACTCGTTATGCATACGGCAGCAAA
GACTTTTGCTTTCCTAGTCTACAAACAATCGCTAAAAAAATGGACAAGACTCCTGTTACAGT
TAGAGGCTACTTGAAACTGCTTGAAAGGTACGGTTTTATTTGGAAGGTAAACGTCCGTAATA
AAACCAAGGATAACACAGAGGAATCCCCGATTTTTAAGATTAGACGTAAGGTTCCTTTGCTT
TCAGAAGAACTTTTAAATGGAAACCCTAATATTGAAATTCCAGATGACGAGGAAGCACATGT
AAAGAAGGCTTTAAAAAAGGAAAAAGAGGGTCTTCCAAAGGTTTTGAAAAAAGAGCACGAT
GAATTTGTTAAAAAAATGATGGATGAGTCAGAAACAATTAATATTCCAGAGGCCTTACAATA
TGACACAATGTATGAAGATATACTCAGTAAAGGAGAAATTCGAAAAGAAATCAAAAAACAAA
TACCTAATCCTACAACATCTTTTGAGAGTATATCAATGACAACTGAAGAGGAAAAAGTCGAC
AGTACTTTAAAAAGCGAAATGCAAAATCGTGTCTCTAAGCCTTCTTTTGATACCTGGTTTAAA
AACACTAAGATCAAAATTGAAAATAAAAATTGTTTATTACTTGTACCGAGTGAATTTGCATTT
GAATGGATTAAGAAAAGATATTTAGAAACAATTAAAACAGTCCTTGAAGAAGCTGGATATGT
TTTCGAAAAAATCGAACTAAGAAAAGTGCAATAAACTGCTGAAGTATTTCAGCAGTTTTTTT
ATTTAGAAATAGTGAAAAAAATATAATCAGGGAGGTATCAATATTTAATGAGTACTGATTTAA
ATTTATTTAGACTGGAATTAATAATTAACACGTAGACTAATTAAAATTTAATGAGGGATAAAG
AGGATACAAAAATATTAATTTCAATCCCTATTAAATTTTAACAAGGGGGGGATTAAAATTTAA
TTAGAGGTTTATCCACAAGAAAAGACCCTAATAAAATTTTTACTAGGGTTATAACACTGATTA
ATTTCTTAATGGGGGAGGGATTAAAATTTAATGACAAAGAAAACAATCTTTTAAGAAAAGCTT
TTAAAAGATAATAATAAAAAGAGCTTTGCGATTAAGCAAAACTCTTTACTTTTTCATTGACAT
TATCAAATTCATCGATTTCAAATTGTTGTTGTATCATAAAGTTAATTCTGTTTTGCACAACCTT
TTCAGGAATATAAAACACATCTGAGGCTTGTTTTATAAACTCAGGGTCGCTAAAGTCAATGT
AACGTAGCATATGATATGGTATAGCTTCCACCCAAGTTAGCCTTTCTGCTTCTTCTGAATGT
TTTTCATATACTTCCATGGGTATCTCTAAATGATTTTCCTCATGTAGCAAGGTATGAGCAAAA
AGTTTATGGAATTGATAGTTCCTCTCTTTTTCTTCAACTTTTTTATCTAAAACAAACACTTTAA
CATCTGAGTCAATGTAAGCATAAGATGTTTTTCCAGTCATAATTTCAATCCCAAATCTTTTAG
ACAGAAATTCTGGACGTAAATCTTTTGGTGAAAGAATTTTTTTATGTAGCAATATATCCGATA
CAGCACCTTCTAAAAGCGTTGGTGAATAGGGCATTTTACCTATCTCCTCTCATTTTGTGGAA
TAAAAATAGTCATATTCGTCCATCTACCTATCCTATTATCGAACAGTTGAACTTTTTAATCAA
GGATCAGTCCTTTTTTTCATTATTCTTAAACTGTGCTCTTAACTTTAACAACTCGCAATCTTAT
CGGGCTATGCATGCTTACATCGTCCGCTGCCACCCAGCCTGGTTTCCAAAGGGAGAAGAA
GCAGAGCTTATAGAAGAAATCATTCAGCAGGTGCTCGACTCCAAAAATATAGATATTAAAAA
ACTTCGCGAGGAAGCGGCGATTATGATGAGCTGCAAAGGCTCCATCAAAGCAAATCGCCA
CCTCAGAAACGACGAAATCAAAGCGCTTCTGGACGACCTCCGAAGCACATCAGACCCATTT
ACATGCCCGCACGGCCGCCCGATCATCATTCACCACTCGACATATGAGATGGAAAAGATGT
TCAAACGCGTGATGTAGCGGGGGTGGTAGGCATTGATCCATTCTCTGAATGGATCAATGCT
TTTTTTGTACTCCTTCGAGTGTCAGATTATAAGAAACGGTTGTAAACGTGTGAGCAGTGTTA

ACGCATTATACTTCATGGTTCCTCCGTATAACAAAATTTCCATTTACAGGCAATTGGGATTAA
TGTATATTTTGGGTGAGGGGGTGGAAACATATGGCATGTGGTCCGAAGATCATTATCTTTTT
GGATGAACTTGATGATCCAAATGTGAGAATCATTTTAGAATGCAGAATTTAAATTACAACATT
GTGTTTTGCTCCCTGTTCGTGTGAACAGGGAGATTTTTTCGTTTATATAACTGGTGATGTCC
AAATATAGGGTGTTATGAATTTTCCATATCTGCCATCGGCTTTAAGCTGCTTCTCTATTAGG
CTGGTTATCAAAAGCAAGTTCAATGATAGCCATGAAAGCTGATATCACAACAAGCAAGACAT
CTGGAATATAAATAGACTGCATAAAGTAATCTGCAAAACAGATCGTTATAAGATTGCTGATA
AAATACAGTATAAAAGCAAAAAGTGCAGTCTTTTTCAATGAAAGTGATTTTGCCAAAATGATA
GAGATAATCTTTGTGAGTGGGTCTATGATTAAACCTACAGCAAAGCAGGCTAAAATAAATAA
AGCTAATGTTTGATTGGAAGCGTATGTGATGCCAAGTATGGAGAATAAGCCTTTCATCCCG
AAGAAAAAGCCGCCGAAGATGACTGCAAGCGCGATGGCCGCGATAAAAGTAATAGATACA
ATTCCGATTAATTTTGATCTCAGGTTAAAATCTTTAGAGTGCTCGTTCTGTTCTTTTTGGTTC
ATGTATGACACTCCCTTCACCTGCTTACTCATACAAACAGCACCCTTGAATCAGCCCAAAAT
ATCTATAAGTCTGTTTTCTGACGAAAATAGTATAGCATATTTTAACAAAACCAAAAAAGGCCC
TTTCCACTGGGGAAGGGCCTTTTTCATCATGTTAACTCCTGAGATTCGCCGAAGCTTTCTCT
TTTTTTGGCCAAATAAAATAGCTGGCTGTAATGAGAAAGTCAATGCCTATAATGACAGTCCA
CAGTTTAAGAATGCCGCTCAACGCCTCGGTTCGGGAAGAGTCATTGATAAAATAGATCATC
CCTGCCAATAGTCCCGCACCAATTAAGTAGGCCAGCACATGTCTTAGCCAGCCTTTCGCAC
CGTGCTTGGCATGGTCCATTCCAAAACGTTTCAGCGGTTTTGTTCCTTTTTTTGTCACATAA
TATTGAAACTTTTCATCTGCCCATTGAATCATTTGTTTCCCATAAGCGATCGATATACCGATG
TAAACAGCTGCAATCCCATGGGCTGCGGTCGCTGAGGCTCCGCGGTAAAGGTCCACACCA
GTTGCCGCTAACAAGATCAAATCGATAACTGGAGTTAAGGCAAGGAATAGCAGTCCTAATG
TATGTCGTTTGAATACGTAGCGTACAGCAAGCCCCAAAACAATGACAACCCAAAACGCAAT
TTCACAGAAAACAATCATCCATGCGATACCGTTCAATCCATTCCCCTCTTTTCAATACAGTT
GTATTATTTAAATAATATGTTACCATCTTTGTTTTTGTAACACAACTGTATTATAAAAATTGCC
CTCTCATTTTTTTCTTGTTACGAATTGGATC

>p14059_pIcBReT-sh-pks500

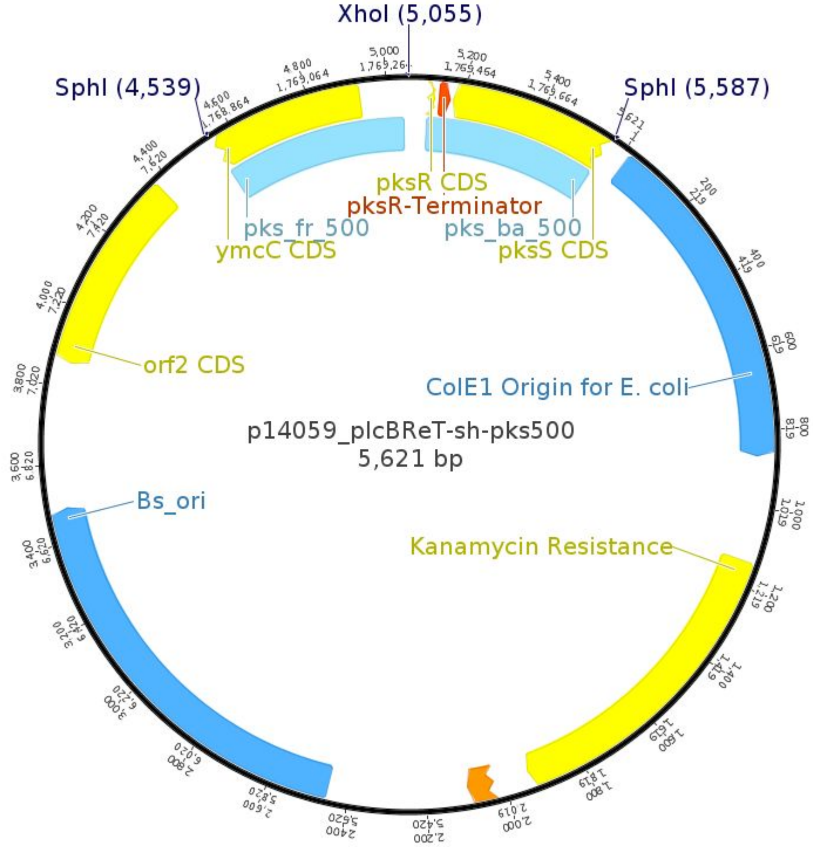

CAGACCCCGTAGAAAAGATCAAAGGATCTTCTTGAGATCCTTTTTTTCTGCGCGTAATCTGC
TGCTTGCAAACAAAAAAACCACCGCTACCAGCGGTGGTTTGTTTGCCGGATCAAGAGCTAC
CAACTCTTTTTCCGAAGGTAACTGGCTTCAGCAGAGCGCAGATACCAAATACTGTTCTTCTA
GTGTAGCCGTAGTTAGGCCACCACTTCAAGAACTCTGTAGCACCGCCTACATACCTCGCTC
TGCTAATCCTGTTACCAGTGGCTGCTGCCAGTGGCGATAAGTCGTGTCTTACCGGGTTGGA
CTCAAGACGATAGTTACCGGATAAGGCGCAGCGGTCGGGCTGAACGGGGGGTTCGTGCA
CACAGCCCAGCTTGGAGCGAACGACCTACACCGAACTGAGATACCTACAGCGTGAGCTAT
GAGAAAGCGCCACGCTTCCCGAAGGGAGAAAGGCGGACAGGTATCCGGTAAGCGGCAGG
GTCGGAACAGGAGAGCGCACGAGGGAGCTTCCAGGGGGAAACGCCTGGTATCTTTATAGT
CCTGTCGGGTTTCGCCACCTCTGACTTGAGCGTCGATTTTTGTGATGCTCGTCAGGGGGG
CGGAGCCTATGGAAAAACGCCAGCAACGCGGCCTTTTTACGGTTCCTGGCCTTTTGCTGG
CCTTTTGCTCACATGTTCTTTCCTGCGTTATCCCCTGATTCTGTGGATAACCGTATTACCGC
CTTTGAGTGAGCTGATACCGCTCGCCGCAGCCGAACGACCGAGCGCAGCGAGTCAGTGA
GCGAGGAAGCGGAAGAGCGCCCAATACGCAAACCGCCTCTCCCCGCGCGTTGGCCGATT
CATTAATGCAGGTTGATCACCTAGAGGATCCCCCAAAGCATAAAAACTTGCATGGACTAAT
GCTTGAAACCCAGGACAATAACCTTATAGCTTGTAAATTCTATCATAATTGTGGTTTCAAAAT
CGGCTCCGTCGATACTATGTTATACGCCAACTTTGAAAACAACTTTGAAAAAGCTGTTTTCT
GGTATTTAAGGTTTTAGAATGCAAGGAACAGTGAATTGGAGTTCGTCTTGTTATAATTAGCT
TCTTGGGGTATCTTTAAATACTGTAGAAAAGAGGAAGGAAATAATAAATGGCTAAAATGAGA
ATATCACCGGAATTGAAAAAACTGATCGAAAAATACCGCTGCGTAAAAGATACGGAAGGAA
TGTCTCCTGCTAAGGTATATAAGCTGGTGGGAGAAAATGAAAACCTATATTTAAAAATGACG

GACAGCCGGTATAAAGGGACCACCTATGATGTGGAACGGGAAAAGGACATGATGCTATGG
CTGGAAGGAAAGCTGCCTGTTCCAAAGGTCCTGCACTTTGAACGGCATGATGGCTGGAGC
AATCTGCTCATGAGTGAGGCCGATGGCGTCCTTTGCTCGGAAGAGTATGAAGATGAACAAA
GCCCTGAAAAGATTATCGAGCTGTATGCGGAGTGCATCAGGCTCTTTCACTCCATCGACAT
ATCGGATTGTCCCTATACGAATAGCTTAGACAGCCGCTTAGCCGAATTGGATTACTTACTGA
ATAACGATCTGGCCGATGTGGATTGCGAAAACTGGGAAGAAGACACTCCATTTAAAGATCC
GCGCGAGCTGTATGATTTTTTAAAGACGGAAAAGCCCGAAGAGGAACTTGTCTTTTCCCAC
GGCGACCTGGGAGACAGCAACATCTTTGTGAAAGATGGCAAAGTAAGTGGCTTTATTGATC
TTGGGAGAAGCGGCAGGGCGGACAAGTGGTATGACATTGCCTTCTGCGTCCGGTCGATCA
GGGAGGATATCGGGGAAGAACAGTATGTCGAGCTATTTTTTGACTTACTGGGGATCAAGCC
TGATTGGGAGAAAATAAAATATTATATTTTACTGGATGAATTGTTTTAGTACCTAGATTTAGA
TGTCTAAAAAGCCTGCAGTCGCGAGGCCTCGACGGATCCCCAAAGCACTTGCATAGGCTA
ATGCCTGGCTTGGTTTGTTGAGACATTACCTGAGAGCCAAAAAACTTTTTATGAATATGAAT
TAAAAAAAAGAACCAACAAAGGCTGAGACAGACTCCAAACGAGTCTGTTTTTTTAAAAAAAA
TATTAGGAGCATTGAATATATATTAGAGAATTAAGAAAGACATGGGAATAAAAATATTTTAAA
TCCAGTAAAAATATGATAAGATTATTTCAGAATATGAAGAACTCTGTTTGTTTTTGATGAAAA
AACAAACAAAAAAAAATCCACCTAACGGAATCTCAATTTAACTAACAGCGGCCAAACTGAGAA
GTTAAATTTGAGAAGGGGAAAAGGCGGATTTATACTTGTATTTAACTATCTCCATTTTAACAT
TTTATTAAACCCCATACAAGTGAAAATCCTCTTTTACACTGTTCCTTTAGGTGATCGCGGAG
GGACATTATGAGTGAAGTAAACCTAAAAGGAAATACAGATGAATTAGTGTATTATCGACAGC
AAACCACTGGAAATAAAATCGCCAGGAAGAGAATCAAAAAAGGGAAAGAAGAAGTTTATTA
TGTTGCTGAAACGGAAGAGAAGATATGGACAGAAGAGCAAATAAAAAACTTTTCTTTAGACA
AATTTGGTACGCATATACCTTACATAGAAGGTCATTATACAATCTTAAATAATTACTTCTTTG
ATTTTTGGGGCTATTTTTTAGGTGCTGAAGGAATTGCGCTCTATGCTCACCTAACTCGTTAT
GCATACGGCAGCAAAGACTTTTGCTTTCCTAGTCTACAAACAATCGCTAAAAAAATGGACAA
GACTCCTGTTACAGTTAGAGGCTACTTGAAACTGCTTGAAAGGTACGGTTTTATTTGGAAG
GTAAACGTCCGTAATAAAACCAAGGATAACACAGAGGAATCCCCGATTTTTAAGATTAGAC
GTAAGGTTCCTTTGCTTTCAGAAGAACTTTTAAATGGAAACCCTAATATTGAAATTCCAGAT
GACGAGGAAGCACATGTAAAGAAGGCTTTAAAAAAGGAAAAAGAGGGTCTTCCAAAGGTTT
TGAAAAAAGAGCACGATGAATTTGTTAAAAAAAATGATGGATGAGTCAGAAACAATTAATATT
CCAGAGGCCTTACAATATGACACAATGTATGAAGATATACTCAGTAAAGGAGAAATTCGAAA
AGAAATCAAAAAACAAATACCTAATCCTACAACATCTTTTGAGAGTATATCAATGACAACTGA
AGAGGAAAAAGTCGACAGTACTTTAAAAAGCGAAATGCAAAATCGTGTCTCTAAGCCTTCTT
TTGATACCTGGTTTAAAAACACTAAGATCAAAATTGAAAATAAAAATTGTTTATTACTTGTAC
CGAGTGAATTTGCATTTGAATGGATTAAGAAAAGATATTTAGAAACAATTAAAACAGTCCTT
GAAGAAGCTGGATATGTTTTCGAAAAAATCGAACTAAGAAAAGTGCAATAAACTGCTGAAGT
ATTTCAGCAGTTTTTTTTATTTAGAAATAGTGAAAAAAATATAATCAGGGAGGTATCAATATT
TAATGAGTACTGATTTAAATTTATTTAGACTGGAATTAATAATTAACACGTAGACTAATTAAAA
TTTAATGAGGGATAAAGAGGATACAAAAATATTAATTTCAATCCCTATTAAATTTTAACAAGG
GGGGGATTAAAATTTAATTAGAGGTTTATCCACAAGAAAAGACCCTAATAAAATTTTTACTAG
GGTTATAACACTGATTAATTTCTTAATGGGGGAGGGATTAAAATTTAATGACAAAGAAAACA
ATCTTTTAAGAAAAGCTTTTAAAAGATAATAATAAAAAGAGCTTTGCGATTAAGCAAAACTCT
TTACTTTTTCATTGACATTATCAAATTCATCGATTTCAAATTGTTGTTGTATCATAAAGTTAAT
TCTGTTTTGCACAACCTTTTCAGGAATATAAAACACATCTGAGGCTTGTTTTATAAACTCAGG
GTCGCTAAAGTCAATGTAACGTAGCATATGATATGGTATAGCTTCCACCCAAGTTAGCCTTT
CTGCTTCTTCTGAATGTTTTTCATATACTTCCATGGGTATCTCTAAATGATTTTCCTCATGTA
GCAAGGTATGAGCAAAAAGTTTATGGAATTGATAGTTCCTCTCTTTTTCTTCAACTTTTTTAT
CTAAAACAAACACTTTAACATCTGAGTCAATGTAAGCATAAGATGTTTTTCCAGTCATAATTT

CAATCCCAAATCTTTTAGACAGAAATTCTGGACGTAAATCTTTTGGTGAAAGAATTTTTTTAT
GTAGCAATATATCCGATACAGCACCTTCTAAAAGCGTTGGTGAATAGGGCATTTTACCTATC
TCCTCTCATTTTGTGGAATAAAAATAGTCATATTCGTCCATCTACCTATCCTATTATCGAACA
GTTGAACTTTTTAATCAAGGATCAGTCCTTTTTTTCATTATTCTTAAACTGTGCTCTTAACTTT
AACAACTCGCAATCTTATCGGGCTATGCATGCTCCCGCACCAATTAAGTAGGCCAGCACAT
GTCTTAGCCAGCCTTTCGCACCGTGCTTGGCATGGTCCATTCCAAAACGTTTCAGCGGTTT
TGTTCCTTTTTTTGTCACATAATATTGAAACTTTTCATCTGCCCATTGAATCATTTGTTTCCCA
TAAGCGATCGATATACCGATGTAAACAGCTGCAATCCCATGGGCTGCGGTCGCTGAGGCT
CCGCGGTAAAGGTCCACACCAGTTGCCGCTAACAAGATCAAATCGATAACTGGAGTTAAGG
CAAGGAATAGCAGTCCTAATGTATGTCGTTTGAATACGTAGCGTACAGCAAGCCCCAAAAC
AATGACAACCCAAAACGCAATTTCACAGAAAACAATCATCCATGCGATACCGTTCAATCCAT
TCCCCTCTTTTCAATACAGTTGTATTATTTAAATAATATGTTACCATCTTTGTTTTTGTAACAC
AACTGTATTATAAAAATTGCCCTCTCATTTTTTTCTTGTTACGAATTGGATCCTCGAGGCCTA
CCGTTCGTATAGCATACATTATACGAACGGTAGGCCTCTAGATGAATTGGTGAAGCGCTGA
GAAAACACAAACGCCCCCTCTTTTAAAAGGGGGCGTTTTGAATGTTATTTTGAAAGTGAAAC
AGGGAGACTTTCTAATCCTCTTAAAAAGACATTTTTTCTCCATTGAATGTCATCAGGTGCAA
CCGCAAGTTCAATATCAGGAAATCTCTTCAAAAGTGCTTTAAATGCAATGTGGCCTTCCAGC
CTGGCAAGAGGCGCTCCTAAGCAGAAATGAATGCCAAAACCAAAAGAAATATGTCTATTAG
GCGACCGATTTATATTTAATATTTCGGGGTTCTCAAAAAAATTCGGGTCGCGATTGGCAGAT
CCGATGCCTATAAAAATCATGTCTCCTCTTTTGATCGAATGCCCCTTATATGTAAAGTCTTC
GATGGCCCACCGATTTGCCATCATAACGACAGGTGAGGTGTATCGCAGCAATTCTTCAACC
GCTGTAGCGATCATTTCAGGCTGCGCATGCACGCGTTGATCAGGCGCCTCGTTCCACTGA
GCGT

>p14060_pIcBReT-sh-pks1k

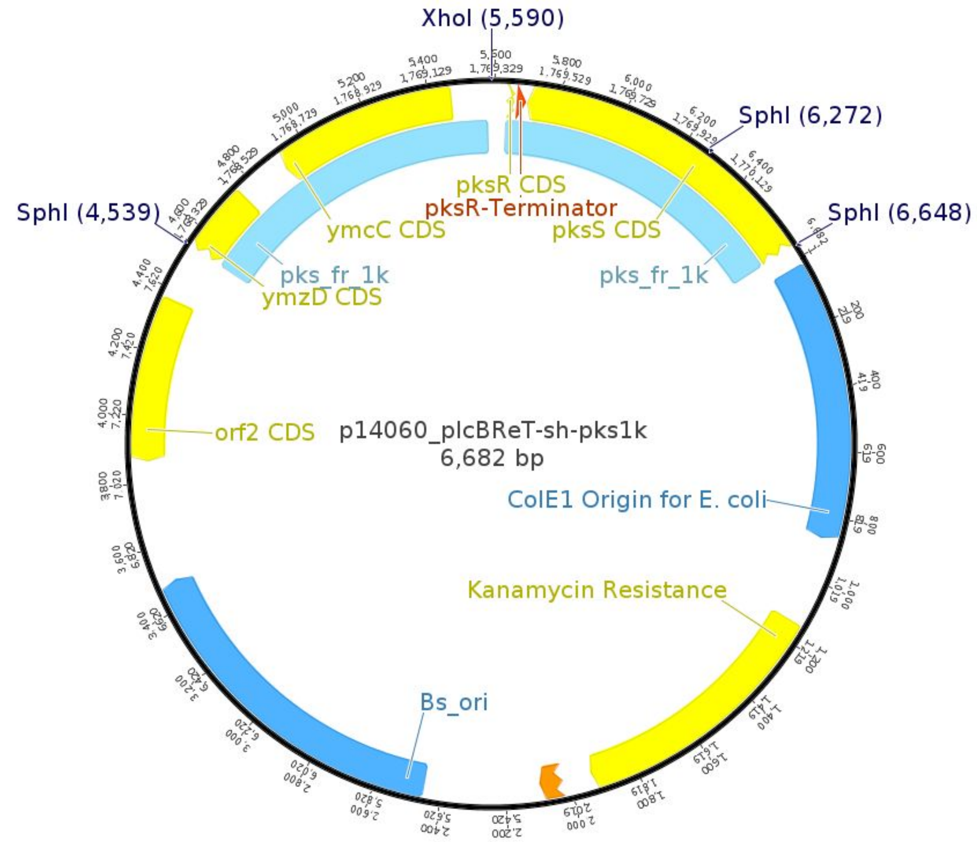

CAGACCCCGTAGAAAAGATCAAAGGATCTTCTTGAGATCCTTTTTTTCTGCGCGTAATCTGC
TGCTTGCAAACAAAAAAACCACCGCTACCAGCGGTGGTTTGTTTGCCGGATCAAGAGCTAC
CAACTCTTTTTCCGAAGGTAACTGGCTTCAGCAGAGCGCAGATACCAAATACTGTTCTTCTA
GTGTAGCCGTAGTTAGGCCACCACTTCAAGAACTCTGTAGCACCGCCTACATACCTCGCTC
TGCTAATCCTGTTACCAGTGGCTGCTGCCAGTGGCGATAAGTCGTGTCTTACCGGGTTGGA
CTCAAGACGATAGTTACCGGATAAGGCGCAGCGGTCGGGCTGAACGGGGGGTTCGTGCA
CACAGCCCAGCTTGGAGCGAACGACCTACACCGAACTGAGATACCTACAGCGTGAGCTAT
GAGAAAGCGCCACGCTTCCCGAAGGGAGAAAGGCGGACAGGTATCCGGTAAGCGGCAGG
GTCGGAACAGGAGAGCGCACGAGGGAGCTTCCAGGGGGAAACGCCTGGTATCTTTATAGT
CCTGTCGGGTTTCGCCACCTCTGACTTGAGCGTCGATTTTTGTGATGCTCGTCAGGGGGG
CGGAGCCTATGGAAAAACGCCAGCAACGCGGCCTTTTTACGGTTCCTGGCCTTTTGCTGG
CCTTTTGCTCACATGTTCTTTCCTGCGTTATCCCCTGATTCTGTGGATAACCGTATTACCGC
CTTTGAGTGAGCTGATACCGCTCGCCGCAGCCGAACGACCGAGCGCAGCGAGTCAGTGA
GCGAGGAAGCGGAAGAGCGCCCAATACGCAAACCGCCTCTCCCCGCGCGTTGGCCGATT
CATTAATGCAGGTTGATCACCTAGAGGATCCCCCAAAGCATAAAAACTTGCATGGACTAAT
GCTTGAAACCCAGGACAATAACCTTATAGCTTGTAAATTCTATCATAATTGTGGTTTCAAAAT
CGGCTCCGTCGATACTATGTTATACGCCAACTTTGAAAACAACTTTGAAAAAGCTGTTTTCT
GGTATTTAAGGTTTTAGAATGCAAGGAACAGTGAATTGGAGTTCGTCTTGTTATAATTAGCT
TCTTGGGGTATCTTTAAATACTGTAGAAAAGAGGAAGGAAATAATAAATGGCTAAAATGAGA
ATATCACCGGAATTGAAAAAACTGATCGAAAAATACCGCTGCGTAAAAGATACGGAAGGAA
TGTCTCCTGCTAAGGTATATAAGCTGGTGGGAGAAAATGAAAACCTATATTTAAAAATGACG
GACAGCCGGTATAAAGGGACCACCTATGATGTGGAACGGGAAAAGGACATGATGCTATGG

CTGGAAGGAAAGCTGCCTGTTCCAAAGGTCCTGCACTTTGAACGGCATGATGGCTGGAGC
AATCTGCTCATGAGTGAGGCCGATGGCGTCCTTTGCTCGGAAGAGTATGAAGATGAACAAA
GCCCTGAAAAGATTATCGAGCTGTATGCGGAGTGCATCAGGCTCTTTCACTCCATCGACAT
ATCGGATTGTCCCTATACGAATAGCTTAGACAGCCGCTTAGCCGAATTGGATTACTTACTGA
ATAACGATCTGGCCGATGTGGATTGCGAAAACTGGGAAGAAGACACTCCATTTAAAGATCC
GCGCGAGCTGTATGATTTTTTAAAGACGGAAAAGCCCGAAGAGGAACTTGTCTTTTCCCAC
GGCGACCTGGGAGACAGCAACATCTTTGTGAAAGATGGCAAAGTAAGTGGCTTTATTGATC
TTGGGAGAAGCGGCAGGGCGGACAAGTGGTATGACATTGCCTTCTGCGTCCGGTCGATCA
GGGAGGATATCGGGGAAGAACAGTATGTCGAGCTATTTTTTGACTTACTGGGGATCAAGCC
TGATTGGGAGAAAATAAAATATTATATTTTACTGGATGAATTGTTTTAGTACCTAGATTTAGA
TGTCTAAAAAGCCTGCAGTCGCGAGGCCTCGACGGATCCCCAAAGCACTTGCATAGGCTA
ATGCCTGGCTTGGTTTGTTGAGACATTACCTGAGAGCCAAAAAACTTTTTATGAATATGAAT
TAAAAAAAAGAACCAACAAAGGCTGAGACAGACTCCAAACGAGTCTGTTTTTTTAAAAAAAA
TATTAGGAGCATTGAATATATATTAGAGAATTAAGAAAGACATGGGAATAAAAATATTTTAAA
TCCAGTAAAAATATGATAAGATTATTTCAGAATATGAAGAACTCTGTTTGTTTTTGATGAAAA
AACAAACAAAAAAAATCCACCTAACGGAATCTCAATTTAACTAACAGCGGCCAAACTGAGAA
GTTAAATTTGAGAAGGGGAAAAGGCGGATTTATACTTGTATTTAACTATCTCCATTTTAACAT
TTTATTAAACCCCATACAAGTGAAAATCCTCTTTTACACTGTTCCTTTAGGTGATCGCGGAG
GGACATTATGAGTGAAGTAAACCTAAAAGGAAATACAGATGAATTAGTGTATTATCGACAGC
AAACCACTGGAAATAAAATCGCCAGGAAGAGAATCAAAAAAGGGAAAGAAGAAGTTTATTA
TGTTGCTGAAACGGAAGAGAAGATATGGACAGAAGAGCAAATAAAAAACTTTTCTTTAGACA
AATTTGGTACGCATATACCTTACATAGAAGGTCATTATACAATCTTAAATAATTACTTCTTTG
ATTTTTGGGGCTATTTTTTAGGTGCTGAAGGAATTGCGCTCTATGCTCACCTAACTCGTTAT
GCATACGGCAGCAAAGACTTTTGCTTTCCTAGTCTACAAACAATCGCTAAAAAAATGGACAA
GACTCCTGTTACAGTTAGAGGCTACTTGAAACTGCTTGAAAGGTACGGTTTTATTTGGAAG
GTAAACGTCCGTAATAAAACCAAGGATAACACAGAGGAATCCCCGATTTTTAAGATTAGAC
GTAAGGTTCCTTTGCTTTCAGAAGAACTTTTAAATGGAAACCCTAATATTGAAATTCCAGAT
GACGAGGAAGCACATGTAAAGAAGGCTTTAAAAAAGGAAAAAGAGGGTCTTCCAAAGGTTT
TGAAAAAAGAGCACGATGAATTTGTTAAAAAAATGATGGATGAGTCAGAAACAATTAATATT
CCAGAGGCCTTACAATATGACACAATGTATGAAGATATACTCAGTAAAGGAGAAATTCGAAA
AGAAATCAAAAAACAAATACCTAATCCTACAACATCTTTTGAGAGTATATCAATGACAACTGA
AGAGGAAAAAGTCGACAGTACTTTAAAAAGCGAAATGCAAAATCGTGTCTCTAAGCCTTCTT
TTGATACCTGGTTTAAAAACACTAAGATCAAAATTGAAAATAAAAATTGTTTATTACTTGTAC
CGAGTGAATTTGCATTTGAATGGATTAAGAAAAGATATTTAGAAACAATTAAAACAGTCCTT
GAAGAAGCTGGATATGTTTTCGAAAAAATCGAACTAAGAAAAGTGCAATAAACTGCTGAAGT
ATTTCAGCAGTTTTTTTTATTTAGAAATAGTGAAAAAAATATAATCAGGGAGGTATCAATATT
TAATGAGTACTGATTTAAATTTATTTAGACTGGAATTAATAATTAACACGTAGACTAATTAAAA
TTTAATGAGGGATAAAGAGGATACAAAAATATTAATTTCAATCCCTATTAAATTTTAACAAGG
GGGGGATTAAAATTTAATTAGAGGTTTATCCACAAGAAAAGACCCTAATAAAATTTTTACTAG
GGTTATAACACTGATTAATTTCTTAATGGGGGAGGGATTAAAATTTAATGACAAAGAAAACA
ATCTTTTAAGAAAAGCTTTTAAAAGATAATAATAAAAAGAGCTTTGCGATTAAGCAAAACTCT
TTACTTTTTCATTGACATTATCAAATTCATCGATTTCAAATTGTTGTTGTATCATAAAGTTAAT
TCTGTTTTGCACAACCTTTTCAGGAATATAAAACACATCTGAGGCTTGTTTTATAAACTCAGG
GTCGCTAAAGTCAATGTAACGTAGCATATGATATGGTATAGCTTCCACCCAAGTTAGCCTTT
CTGCTTCTTCTGAATGTTTTTCATATACTTCCATGGGTATCTCTAAATGATTTTCCTCATGTA
GCAAGGTATGAGCAAAAAGTTTATGGAATTGATAGTTCCTCTCTTTTTCTTCAACTTTTTTAT
CTAAAACAAACACTTTAACATCTGAGTCAATGTAAGCATAAGATGTTTTTCCAGTCATAATTT
CAATCCCAAATCTTTTAGACAGAAATTCTGGACGTAAATCTTTTGGTGAAAGAATTTTTTTAT

GTAGCAATATATCCGATACAGCACCTTCTAAAAGCGTTGGTGAATAGGGCATTTTACCTATC
TCCTCTCATTTTGTGGAATAAAAATAGTCATATTCGTCCATCTACCTATCCTATTATCGAACA
GTTGAACTTTTTAATCAAGGATCAGTCCTTTTTTTCATTATTCTTAAACTGTGCTCTTAACTTT
AACAACTCGCAATCTTATCGGGCTATGCATGCACCTACAGCAAAGCAGGCTAAAATAAATA
AAGCTAATGTTTGATTGGAAGCGTATGTGATGCCAAGTATGGAGAATAAGCCTTTCATCCC
GAAGAAAAAGCCGCCGAAGATGACTGCAAGCGCGATGGCCGCGATAAAAGTAATAGATAC
AATTCCGATTAATTTTGATCTCAGGTTAAAATCTTTAGAGTGCTCGTTCTGTTCTTTTTGGTT
CATGTATGACACTCCCTTCACCTGCTTACTCATACAAACAGCACCCTTGAATCAGCCCAAAA
TATCTATAAGTCTGTTTTCTGACGAAAATAGTATAGCATATTTTAACAAAACCAAAAAAGGCC
CTTTCCACTGGGGAAGGGCCTTTTTCATCATGTTAACTCCTGAGATTCGCCGAAGCTTTCTC
TTTTTTTGGCCAAATAAAATAGCTGGCTGTAATGAGAAAGTCAATGCCTATAATGACAGTCC
ACAGTTTAAGAATGCCGCTCAACGCCTCGGTTCGGGAAGAGTCATTGATAAAATAGATCAT
CCCTGCCAATAGTCCCGCACCAATTAAGTAGGCCAGCACATGTCTTAGCCAGCCTTTCGCA
CCGTGCTTGGCATGGTCCATTCCAAAACGTTTCAGCGGTTTTGTTCCTTTTTTTGTCACATA
ATATTGAAACTTTTCATCTGCCCATTGAATCATTTGTTTCCCATAAGCGATCGATATACCGAT
GTAAACAGCTGCAATCCCATGGGCTGCGGTCGCTGAGGCTCCGCGGTAAAGGTCCACACC
AGTTGCCGCTAACAAGATCAAATCGATAACTGGAGTTAAGGCAAGGAATAGCAGTCCTAAT
GTATGTCGTTTGAATACGTAGCGTACAGCAAGCCCCAAAACAATGACAACCCAAAACGCAA
TTTCACAGAAAACAATCATCCATGCGATACCGTTCAATCCATTCCCCTCTTTTCAATACAGTT
GTATTATTTAAATAATATGTTACCATCTTTGTTTTTGTAACACAACTGTATTATAAAAATTGCC
CTCTCATTTTTTTCTTGTTACGAATTGGATCCTCGAGGCCTACCGTTCGTATAGCATACATTA
TACGAACGGTAGGCCTCTAGATGAATTGGTGAAGCGCTGAGAAAACACAAACGCCCCCTC
TTTTAAAAGGGGGCGTTTTGAATGTTATTTTGAAAGTGAAACAGGGAGACTTTCTAATCCTC
TTAAAAAGACATTTTTTCTCCATTGAATGTCATCAGGTGCAACCGCAAGTTCAATATCAGGA
AATCTCTTCAAAAGTGCTTTAAATGCAATGTGGCCTTCCAGCCTGGCAAGAGGCGCTCCTA
AGCAGAAATGAATGCCAAAACCAAAAGAAATATGTCTATTAGGCGACCGATTTATATTTAAT
ATTTCGGGGTTCTCAAAAAAATTCGGGTCGCGATTGGCAGATCCGATGCCTATAAAAATCA
TGTCTCCTCTTTTGATCGAATGCCCCTTATATGTAAAGTCTTCGATGGCCCACCGATTTGCC
ATCATAACGACAGGTGAGGTGTATCGCAGCAATTCTTCAACCGCTGTAGCGATCATTTCAG
GCTGCTGCTTGAGCTTCTCACATTCCTTCTTGTGCTGCAGCAATGCGAGGGTGCCTGAGCC
GAGTAAGTTAACAGTTGTTTCAAGGCCGGCTACAACGAGCAAGAACAGCATCGAATAGAGC
TCTTTTTCGCTTAACTTGCTGCCGTTTTCCTCAGCATGCACAAGTTTGCTGATTAAATCGTCT
TTTGGCTTTATTCTTCTGTCATGGATCAGCTTAGCGATATAATCTTTAAATTCACGAAGGGC
CTGATTTGTCAGCTCTCTATTACCTTCAGAGGTATCAACCATCGCATTGGTCCAGATTTGAA
ACTGTGACCGATCTTCTTTTGGGATTCCCATCAATTCAGATATAACAATAAAAGGCAAAGGG
GAAGCGAAGGATTTCATGATATCCGCTTTATTTTCTTTTTCCATTTCATCTAAAAGCTGTTCA
GCAATTTGTTCAATGCTGCCGCGCAGATTTTCAATGGTTCGGGGAGTAAATGCTTGATGAA
CAAGTGATCTCAGGCGGGTATGGTCAGGTGTGTCTTGCATGCACGCGTTGATCAGGCGCC
TCGTTCCACTGAGCGT

>p14067_pJET-GFPmut2

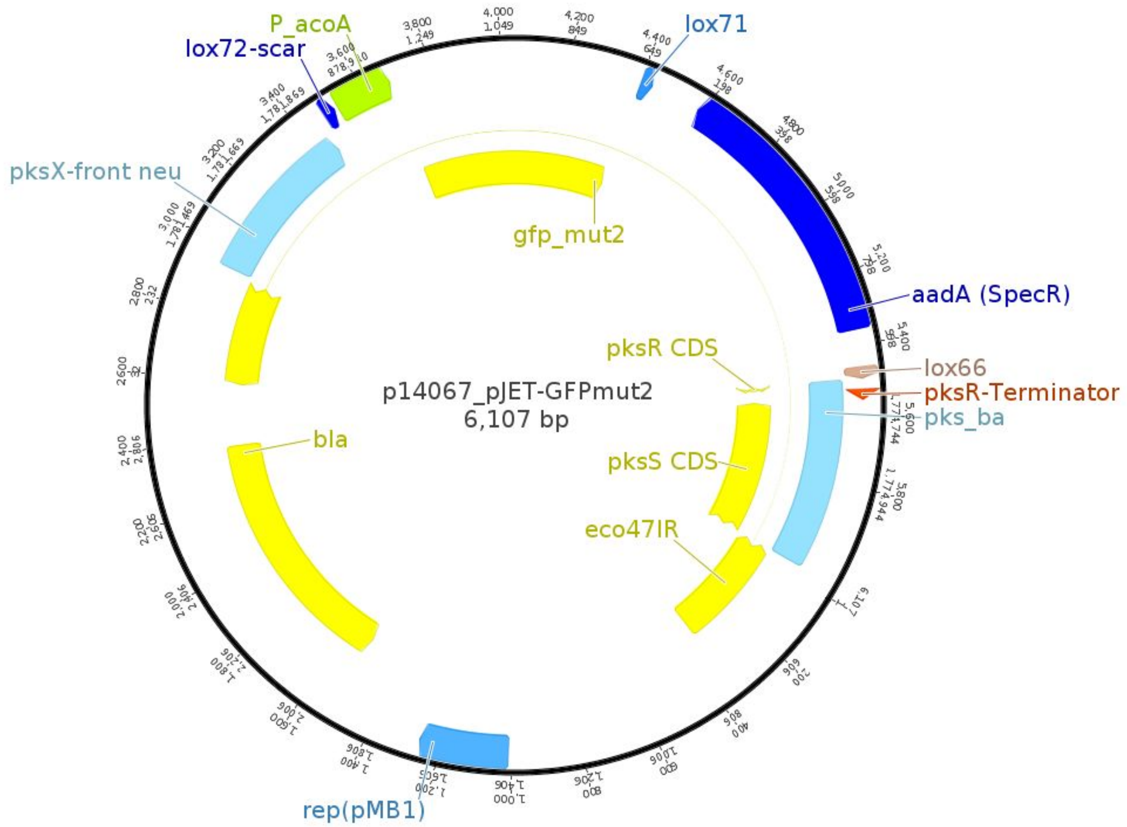

GCCATGGAAAATCGATGTTCTTCTTTTATTCTCTCAAGATTTTCAGGCTGTATATTAAAACTT
ATATTAAGAACTATGCTAACCACCTCATCAGGAACCGTTGTAGGTGGCGTGGGTTTTCTTG
GCAATCGACTCTCATGAAAACTACGAGCTAAATATTCAATATGTTCCTCTTGACCAACTTTAT
TCTGCATTTTTTTTGAACGAGGTTTAGAGCAAGCTTCAGGAAACTGAGACAGGAATTTTATT
AAAAATTTAAATTTTGAAGAAAGTTCAGGGTTAATAGCATCCATTTTTTTGCTTTGCAAGTTCC
TCAGCATTCTTAACAAAAGACGTCTCTTTTGACATGTTTAAAGTTTAAACCTCCTGTGTGAAA
TTATTATCCGCTCATAATTCCACACATTATACGAGCCGGAAGCATAAAGTGTAAAGCCTGGG
GTGCCTAATGAGTGAGCTAACTCACATTAATTGCGTTGCGCTCACTGCCAATTGCTTTCCA
GTCGGGAAACCTGTCGTGCCAGCTGCATTAATGAATCGGCCAACGCGCGGGGAGAGGCG
GTTTGCGTATTGGGCGCTCTTCCGCTTCCTCGCTCACTGACTCGCTGCGCTCGGTCGTTC
GGCTGCGGCGAGCGGTATCAGCTCACTCAAAGGCGGTAATACGGTTATCCACAGAATCAG
GGGATAACGCAGGAAAGAACATGTGAGCAAAAGGCCAGCAAAAGGCCAGGAACCGTAAAA
AGGCCGCGTTGCTGGCGTTTTTCCATAGGCTCCGCCCCCCTGACGAGCATCACAAAAATC
GACGCTCAAGTCAGAGGTGGCGAAACCCGACAGGACTATAAAGATACCAGGCGTTTCCCC
CTGGAAGCTCCCTCGTGCGCTCTCCTGTTCCGACCCTGCCGCTTACCGGATACCTGTCCG
CCTTTCTCCCTTCGGGAAGCGTGGCGCTTTCTCATAGCTCACGCTGTAGGTATCTCAGTTC
GGTGTAGGTCGTTCGCTCCAAGCTGGGCTGTGTGCACGAACCCCCCGTTCAGCCCGACC
GCTGCGCCTTATCCGGTAACTATCGTCTTGAGTCCAACCCGGTAAGACACGACTTATCGCC
ACTGGCAGCAGCCACTGGTAACAGGATTAGCAGAGCGAGGTATGTAGGCGGTGCTACAGA
GTTCTTGAAGTGGTGGCCTAACTACGGCTACACTAGAAGGACAGTATTTGGTATCTGCGCT
CTGCTGAAGCCAGTTACCTTCGGAAAAAGAGTTGGTAGCTCTTGATCCGGCAAACAAACCA

CCGCTGGTAGCGGTGGTTTTTTTGTTTGCAAGCAGCAGATTACGCGCAGAAAAAAAGGATC
TCAAGAAGATCCTTTGATCTTTTCTACGGGGTCTGACGCTCAGTGGAACGAAAACTCACGT
TAAGGGATTTTGGTCATGAGATTATCAAAAAGGATCTTCACCTAGATCCTTTTAAATTAAAAA
TGAAGTTTTAAATCAATCTAAAGTATATATGAGTAAACTTGGTCTGACAGTTACCAATGCTTA
ATCAGTGAGGCACCTATCTCAGCGATCTGTCTATTTCGTTCATCCATAGTTGCCTGACTCCC
CGTCGTGTAGATAACTACGATACGGGAGGGCTTACCATCTGGCCCCAGTGCTGCAATGAT
ACCGCGAGACCCACGCTCACCGGCTCCAGATTTATCAGCAATAAACCAGCCAGCCGGAAG
GGCCGAGCGCAGAAGTGGTCCTGCAACTTTATCCGCCTCCATCCAGTCTATTAATTGTTGC
CGGGAAGCTAGAGTAAGTAGTTCGCCAGTTAATAGTTTGCGCAACGTTGTTGCCATTGCTA
CAGGCATCGTGGTGTCACGCTCGTCGTTTGGTATGGCTTCATTCAGCTCCGGTTCCCAACG
ATCAAGGCGAGTTACATGATCCCCCATGTTGTGCAAAAAAGCGGTTAGCTCCTTCGGTCCT
CCGATCGTTGTCAGAAGTAAGTTGGCCGCAGTGTTATCACTCATGGTTATGGCAGCACTGC
ATAATTCTCTTACTGTCATGCCATCCGTAAGATGCTTTTCTGTGACTGGTGAGTACTCAACC
AAGTCATTCTGAGAATAGTGTATGCGGCGACCGAGTTGCTCTTGCCCGGCGTCAATACGG
GATAATACCGCGCCACATAGCAGAACTTTAAAAGTGCTCATCATTGGAAAACGTTCTTCGG
GGCGAAAACTCTCAAGGATCTTACCGCTGTTGAGATCCAGTTCGATGTAACCCACTCGTGC
ACCCAACTGATCTTCAGCATCTTTTACTTTCACCAGCGTTTCTGGGTGAGCAAAAACAGGAA
GGCAAAATGCCGCAAAAAAGGGAATAAGGGCGACACGGAAATGTTGAATACTCATACTCTT
CCTTTTTCAATATTATTGAAGCATTTATCAGGGTTATTGTCTCATGAGCGGATACATATTTGA
ATGTATTTAGAAAAATAAACAAATAGGGGTTCCGCGCACATTTCCCCGAAAAGTGCCACCT
GACGTCTAAGAAACCATTATTATCATGACATTAACCTATAAAAATAGGCGTATCACGAGGCC
GCCCCTGCAGCCGAATTATATTATTTTTGCCAAATAATTTTTAACAAAAGCTCTGAAGTCTTC
TTCATTTAAATTCTTAGATGATACTTCATCTGGAAAATTGTCCCAATTAGTAGCATCACGCTG
TGAGTAAGTTCTAAACCATTTTTTTATTGTTGTATTATCTCTAATCTTACTACTCGATGAGTTT
TCGGTATTATCTCTATTTTTAACTTGGAGCAGGTTCCATTCATTGTTTTTTTCATCATAGTGA
ATAAAATCAACTGCTTTAACACTTGTGCCTGAACACCATATCCATCCGGCGTAATACGACTC
ACTATAGGGAGAGCGGCCGCCAGATCTTCCGGATGGCTCGAGTTTTTCAGCAAGATCATC
CCTGCCAATAGTCCCGCACCAATTAAGTAGGCCAGCACATGTCTTAGCCAGCCTTTCGCAC
CGTGCTTGGCATGGTCCATTCCAAAACGTTTCAGCGGTTTTGTTCCTTTTTTTGTCACATAA
TATTGAAACTTTTCATCTGCCCATTGAATCATTTGTTTCCCATAAGCGATCGATATACCGATG
TAAACAGCTGCAATCCCATGGGCTGCGGTCGCTGAGGCTCCGCGGTAAAGGTCCACACCA
GTTGCCGCTAACAAGATCAAATCGATAACTGGAGTTAAGGCAAGGAATAGCAGTCCTAATG
TATGTCGTTTGAATACGTAGCGTACAGCAAGCCCCAAAACAATGACAACCCAAAACGCAAT
TTCACAGAAAACAATCATCCATGCGATACCGTTCAATCCATTCCCCTCTTTTCAATACAGTT
GTATTATTTAAATAATATGTTACCATCTTTGTTTTTGTAACACAACTGTATTATAAAAATTGCC
CTCTCATTTTTTTCTTGTTACGAATTGGATCCTCGAGGCCTACCGTTCGTATAGCATACATTA
TACGAACGGTAGGCCTCTAGAGCATGCTGTAAAACGAGACAAATGAATCAGTTTGAGACAA
AACGAGACACACGTCTCAAACTGTCTCCAAAGTGAAGATGAGAAGACTGATTTTACGGGCT
CAAAAGACTGGCACACTTCTTGCATTTATAATGGTGAACCCTAAATAGAAGGAGGCGCACA
AAATGAGTAAAGGAGAAGAACTTTTCACTGGAGTTGTCCCAATTCTTGTTGAATTAGATGGT
GATGTTAATGGGCACAAATTTTCTGTCAGTGGAGAGGGTGAAGGTGATGCAACATACGGAA
AACTTACCCTTAAATTTATTTGCACTACTGGAAAACTACCTGTTCCATGGCCAACACTTGTC
ACTACTTTCGCGTATGGTCTTCAATGCTTTGCGAGATACCCAGATCATATGAAACAGCATGA
CTTTTTCAAGAGTGCCATGCCCGAAGGTTATGTACAGGAAAGAACTATATTTTTCAAAGATG
ACGGGAACTACAAGACACGTGCTGAAGTCAAGTTTGAAGGTGATACCCTTGTTAATAGAAT
CGAGTTAAAAGGTATTGATTTTAAAGAAGATGGAAACATTCTTGGACACAAATTGGAATACA
ACTATAACTCACACAATGTATACATCATGGCAGACAAACAAAAGAATGGAATCAAAGTTAAC
TTCAAAATTAGACACAACATTGAAGATGGAAGCGTTCAACTAGCAGACCATTATCAACAAAA

TACTCCAATTGGCGATGGCCCTGTCCTTTTACCAGACAACCATTACCTGTCCACACAATCTG
CCCTTTCGAAAGATCCCAACGAAAAGAGAGACCACATGGTCCTTCTTGAGTTTGTAACAGC
TGCTGGGATTACACATGGCATGGATGAACTATACAAATAGTACCGTTCGTATAGCATACATT
ATACGAAGTTATTAGACCGGTACTAAATTAAAGTAATAAAGCGTTCTCTAATTTCACAAGAG
GACGCTTTATTCTTCCCAAAAATTGTTCAATATTTATCAATAAATCAGTAGTTTTAAAAGTAA
GCACCTGTTATTGCAATAAAATTAGCCTAATTGAGAGAAGTTTCTATAGAATTTTTCATATAC
TTAACGAGTGCTTTCACCTTTGAATATAGTCCTTCCCACTTATCATCACACTCTCCCCGATA
GCCTTTTCTAGCTATATCCAGTAAAGTTACATGCTCTTTAGGTAAAAGAGGTATAGCCCATT
CTGCAGCGACATCTTTCGAGGTAATTTCACCAGTAGTCACTGTTTGCCACATTCGAGCTAG
GGTTAAAATTACATTACGCTCATCACCTTTTATCCCCTCAATTAGTTCTGGCAAAGAATCCTT
AATTGCTCTTCGAATATCTGTCAAAGGTACGGAGACAAGTATACTTGAAGAATCAGGACCAA
ATAGAGAAATACTATTCTTTCTTGCTTGTGCTAAAACAATAGCCAAATCAGGATCATAGCTT
GGTTCCTGAATTTGTCCATTCTCAAATTCACCCCTGAGCCACTCACCGTATATAAATTCTCT
TTTTGGAGGATATTGCCAAGGGACAACTTCACTCCTATTTATAACCGTAACTTCAAGTGGTC
TAACAGAATCCGTATTTCCAATCTTTCCTGATATAGTCATTAGTCTTTCTGTTAGTTTTTTTCG
AGTTAATTGAGGTAAACTATGATTCACGACGACTAGAACATCTACATCGCTGTTAATGCGTA
AACCACCATTTACTGCTGAACCAAATAGATATACTCCAACTATTGAACTTCCAAATAAATCTT
TTACGATTTTTAATGTTTGAATCGCTTGATTTGGTATTTTTCCGTTAATCAAATTGCTCATGAT
TTCACCTCGTTGATTATGTTCATATAAAGTTTATATTGATACTCAATTTACTTACCCTAGATTG
GACATATACTTAAATTACTGTTCAATAAAGCTGACCATAACTTCGTATAGCATACATTATACG
AACGGTAGGATGAATTGGTGAAGCGCTGAGAAAACACAAACGCCCCCTCTTTTAAAAGGG
GGCGTTTTGAATGTTATTTTGAAAGTGAAACAGGGAGACTTTCTAATCCTCTTAAAAAGACA
TTTTTTCTCCATTGAATGTCATCAGGTGCAACCGCAAGTTCAATATCAGGAAATCTCTTCAA
AAGTGCTTTAAATGCAATGTGGCCTTCCAGCCTGGCAAGAGGCGCTCCTAAGCAGAAATGA
ATGCCAAAACCAAAAGAAATATGTCTATTAGGCGACCGATTTATATTTAATATTTCGGGGTT
CTCAAAAAAATTCGGGTCGCGATTGGCAGATCCGATGCCTATAAAAATCATGTCTCCTCTTT
TGATCGAATGCCCCTTATATGTAAAGTCTTCGATGGCCCACCGATTTGCCATCATAACGACA
GGTGAGGTGTATCGCAGCAATTCTTCAACCGCTGTAGCGATCATTTCAGGCTGCTGCTTGA
GCTTCTCACATTCCTTCTTGTGCTGCAGCAATGCGAGGGTGCCTGAGCCGAGTAAGTTAAC
AGTTGTTTCAAGGCCGGCTACAACGAGCAAGATCTTTCTAGAAGATCTCCTACAATATTCTC
AGCT

>p14071_pIcBReT-sh-pks500-GFPmut2

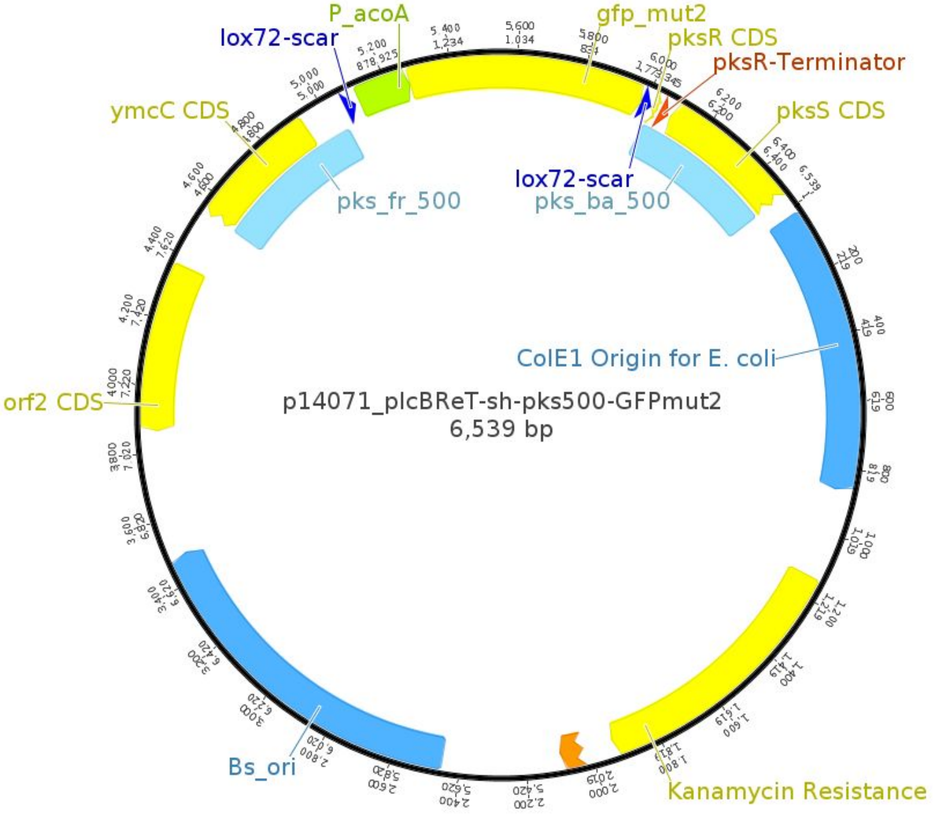

CAGACCCCGTAGAAAAGATCAAAGGATCTTCTTGAGATCCTTTTTTTCTGCGCGTAATCTGC
TGCTTGCAAACAAAAAAACCACCGCTACCAGCGGTGGTTTGTTTGCCGGATCAAGAGCTAC
CAACTCTTTTTCCGAAGGTAACTGGCTTCAGCAGAGCGCAGATACCAAATACTGTTCTTCTA
GTGTAGCCGTAGTTAGGCCACCACTTCAAGAACTCTGTAGCACCGCCTACATACCTCGCTC
TGCTAATCCTGTTACCAGTGGCTGCTGCCAGTGGCGATAAGTCGTGTCTTACCGGGTTGGA
CTCAAGACGATAGTTACCGGATAAGGCGCAGCGGTCGGGCTGAACGGGGGGTTCGTGCA
CACAGCCCAGCTTGGAGCGAACGACCTACACCGAACTGAGATACCTACAGCGTGAGCTAT
GAGAAAGCGCCACGCTTCCCGAAGGGAGAAAGGCGGACAGGTATCCGGTAAGCGGCAGG
GTCGGAACAGGAGAGCGCACGAGGGAGCTTCCAGGGGGAAACGCCTGGTATCTTTATAGT
CCTGTCGGGTTTCGCCACCTCTGACTTGAGCGTCGATTTTTGTGATGCTCGTCAGGGGGG
CGGAGCCTATGGAAAAACGCCAGCAACGCGGCCTTTTTACGGTTCCTGGCCTTTTGCTGG
CCTTTTGCTCACATGTTCTTTCCTGCGTTATCCCCTGATTCTGTGGATAACCGTATTACCGC
CTTTGAGTGAGCTGATACCGCTCGCCGCAGCCGAACGACCGAGCGCAGCGAGTCAGTGA
GCGAGGAAGCGGAAGAGCGCCCAATACGCAAACCGCCTCTCCCCGCGCGTTGGCCGATT
CATTAATGCAGGTTGATCACCTAGAGGATCCCCCAAAGCATAAAAACTTGCATGGACTAAT
GCTTGAAACCCAGGACAATAACCTTATAGCTTGTAAATTCTATCATAATTGTGGTTTCAAAAT
CGGCTCCGTCGATACTATGTTATACGCCAACTTTGAAAACAACTTTGAAAAAGCTGTTTTCT
GGTATTTAAGGTTTTAGAATGCAAGGAACAGTGAATTGGAGTTCGTCTTGTTATAATTAGCT
TCTTGGGGTATCTTTAAATACTGTAGAAAAGAGGAAGGAAATAATAAATGGCTAAAATGAGA
ATATCACCGGAATTGAAAAAACTGATCGAAAAATACCGCTGCGTAAAAGATACGGAAGGAA
TGTCTCCTGCTAAGGTATATAAGCTGGTGGGAGAAAATGAAAACCTATATTTAAAAATGACG
GACAGCCGGTATAAAGGGACCACCTATGATGTGGAACGGGAAAAGGACATGATGCTATGG

CTGGAAGGAAAGCTGCCTGTTCCAAAGGTCCTGCACTTTGAACGGCATGATGGCTGGAGC
AATCTGCTCATGAGTGAGGCCGATGGCGTCCTTTGCTCGGAAGAGTATGAAGATGAACAAA
GCCCTGAAAAGATTATCGAGCTGTATGCGGAGTGCATCAGGCTCTTTCACTCCATCGACAT
ATCGGATTGTCCCTATACGAATAGCTTAGACAGCCGCTTAGCCGAATTGGATTACTTACTGA
ATAACGATCTGGCCGATGTGGATTGCGAAAACTGGGAAGAAGACACTCCATTTAAAGATCC
GCGCGAGCTGTATGATTTTTTAAAGACGGAAAAGCCCGAAGAGGAACTTGTCTTTTCCCAC
GGCGACCTGGGAGACAGCAACATCTTTGTGAAAGATGGCAAAGTAAGTGGCTTTATTGATC
TTGGGAGAAGCGGCAGGGCGGACAAGTGGTATGACATTGCCTTCTGCGTCCGGTCGATCA
GGGAGGATATCGGGGAAGAACAGTATGTCGAGCTATTTTTTGACTTACTGGGGATCAAGCC
TGATTGGGAGAAAATAAAATATTATATTTTACTGGATGAATTGTTTTAGTACCTAGATTTAGA
TGTCTAAAAAGCCTGCAGTCGCGAGGCCTCGACGGATCCCCAAAGCACTTGCATAGGCTA
ATGCCTGGCTTGGTTTGTTGAGACATTACCTGAGAGCCAAAAAACTTTTTATGAATATGAAT
TAAAAAAAAGAACCAACAAAGGCTGAGACAGACTCCAAACGAGTCTGTTTTTTTAAAAAAAA
TATTAGGAGCATTGAATATATATTAGAGAATTAAGAAAGACATGGGAATAAAAATATTTTAAA
TCCAGTAAAAATATGATAAGATTATTTCAGAATATGAAGAACTCTGTTTGTTTTTGATGAAAA
AACAAACAAAAAAAATCCACCTAACGGAATCTCAATTTAACTAACAGCGGCCAAACTGAGAA
GTTAAATTTGAGAAGGGGAAAAGGCGGATTTATACTTGTATTTAACTATCTCCATTTTAACAT
TTTATTAAACCCCATACAAGTGAAAATCCTCTTTTACACTGTTCCTTTAGGTGATCGCGGAG
GGACATTATGAGTGAAGTAAACCTAAAAGGAAATACAGATGAATTAGTGTATTATCGACAGC
AAACCACTGGAAATAAAATCGCCAGGAAGAGAATCAAAAAAGGGAAAGAAGAAGTTTATTA
TGTTGCTGAAACGGAAGAGAAGATATGGACAGAAGAGCAAATAAAAAACTTTTCTTTAGACA
AATTTGGTACGCATATACCTTACATAGAAGGTCATTATACAATCTTAAATAATTACTTCTTTG
ATTTTTGGGGCTATTTTTTAGGTGCTGAAGGAATTGCGCTCTATGCTCACCTAACTCGTTAT
GCATACGGCAGCAAAGACTTTTGCTTTCCTAGTCTACAAACAATCGCTAAAAAAATGGACAA
GACTCCTGTTACAGTTAGAGGCTACTTGAAACTGCTTGAAAGGTACGGTTTTATTTGGAAG
GTAAACGTCCGTAATAAAACCAAGGATAACACAGAGGAATCCCCGATTTTTAAGATTAGAC
GTAAGGTTCCTTTGCTTTCAGAAGAACTTTTAAATGGAAACCCTAATATTGAAATTCCAGAT
GACGAGGAAGCACATGTAAAGAAGGCTTTAAAAAAGGAAAAAGAGGGTCTTCCAAAGGTTT
TGAAAAAAGAGCACGATGAATTTGTTAAAAAAATGATGGATGAGTCAGAAACAATTAATATT
CCAGAGGCCTTACAATATGACACAATGTATGAAGATATACTCAGTAAAGGAGAAATTCGAAA
AGAAATCAAAAAACAAATACCTAATCCTACAACATCTTTTGAGAGTATATCAATGACAACTGA
AGAGGAAAAAGTCGACAGTACTTTAAAAAGCGAAATGCAAAATCGTGTCTCTAAGCCTTCTT
TTGATACCTGGTTTAAAAACACTAAGATCAAAATTGAAAATAAAAATTGTTTATTACTTGTAC
CGAGTGAATTTGCATTTGAATGGATTAAGAAAAGATATTTAGAAACAATTAAAACAGTCCTT
GAAGAAGCTGGATATGTTTTCGAAAAAATCGAACTAAGAAAAGTGCAATAAACTGCTGAAGT
ATTTCAGCAGTTTTTTTTATTTAGAAATAGTGAAAAAAATATAATCAGGGAGGTATCAATATT
TAATGAGTACTGATTTAAATTTATTTAGACTGGAATTAATAATTAACACGTAGACTAATTAAAA
TTTAATGAGGGATAAAGAGGATACAAAAATATTAATTTCAATCCCTATTAAATTTTAACAAGG
GGGGGATTAAAATTTAATTAGAGGTTTATCCACAAGAAAAGACCCTAATAAAATTTTTACTAG
GGTTATAACACTGATTAATTTCTTAATGGGGGAGGGATTAAAATTTAATGACAAAGAAAACA
ATCTTTTAAGAAAAGCTTTTAAAAGATAATAATAAAAAGAGCTTTGCGATTAAGCAAAACTCT
TTACTTTTTCATTGACATTATCAAATTCATCGATTTCAAATTGTTGTTGTATCATAAAGTTAAT
TCTGTTTTGCACAACCTTTTCAGGAATATAAAACACATCTGAGGCTTGTTTTATAAACTCAGG
GTCGCTAAAGTCAATGTAACGTAGCATATGATATGGTATAGCTTCCACCCAAGTTAGCCTTT
CTGCTTCTTCTGAATGTTTTTCATATACTTCCATGGGTATCTCTAAATGATTTTCCTCATGTA
GCAAGGTATGAGCAAAAAGTTTATGGAATTGATAGTTCCTCTCTTTTTCTTCAACTTTTTTAT
CTAAAACAAACACTTTAACATCTGAGTCAATGTAAGCATAAGATGTTTTTCCAGTCATAATTT
CAATCCCAAATCTTTTAGACAGAAATTCTGGACGTAAATCTTTTGGTGAAAGAATTTTTTTAT

GTAGCAATATATCCGATACAGCACCTTCTAAAAGCGTTGGTGAATAGGGCATTTTACCTATC
TCCTCTCATTTTGTGGAATAAAAATAGTCATATTCGTCCATCTACCTATCCTATTATCGAACA
GTTGAACTTTTTAATCAAGGATCAGTCCTTTTTTTCATTATTCTTAAACTGTGCTCTTAACTTT
AACAACTCGCAATCTTATCGGGCTATGCATGCTCCCGCACCAATTAAGTAGGCCAGCACAT
GTCTTAGCCAGCCTTTCGCACCGTGCTTGGCATGGTCCATTCCAAAACGTTTCAGCGGTTT
TGTTCCTTTTTTTGTCACATAATATTGAAACTTTTCATCTGCCCATTGAATCATTTGTTTCCCA
TAAGCGATCGATATACCGATGTAAACAGCTGCAATCCCATGGGCTGCGGTCGCTGAGGCT
CCGCGGTAAAGGTCCACACCAGTTGCCGCTAACAAGATCAAATCGATAACTGGAGTTAAGG
CAAGGAATAGCAGTCCTAATGTATGTCGTTTGAATACGTAGCGTACAGCAAGCCCCAAAAC
AATGACAACCCAAAACGCAATTTCACAGAAAACAATCATCCATGCGATACCGTTCAATCCAT
TCCCCTCTTTTCAATACAGTTGTATTATTTAAATAATATGTTACCATCTTTGTTTTTGTAACAC
AACTGTATTATAAAAATTGCCCTCTCATTTTTTTCTTGTTACGAATTGGATCCTCGAGGCCTA
CCGTTCGTATAGCATACATTATACGAACGGTAGGCCTCTAGAGCATGCTGTAAAACGAGAC
AAATGAATCAGTTTGAGACAAAACGAGACACACGTCTCAAACTGTCTCCAAAGTGAAGATG
AGAAGACTGATTTTACGGGCTCAAAAGACTGGCACACTTCTTGCATTTATAATGGTGAACCC
TAAATAGAAGGAGGCGCACAAAATGAGTAAAGGAGAAGAACTTTTCACTGGAGTTGTCCCA
ATTCTTGTTGAATTAGATGGTGATGTTAATGGGCACAAATTTTCTGTCAGTGGAGAGGGTGA
AGGTGATGCAACATACGGAAAACTTACCCTTAAATTTATTTGCACTACTGGAAAACTACCTG
TTCCATGGCCAACACTTGTCACTACTTTCGCGTATGGTCTTCAATGCTTTGCGAGATACCCA
GATCATATGAAACAGCATGACTTTTTCAAGAGTGCCATGCCCGAAGGTTATGTACAGGAAA
GAACTATATTTTTCAAAGATGACGGGAACTACAAGACACGTGCTGAAGTCAAGTTTGAAGG
TGATACCCTTGTTAATAGAATCGAGTTAAAAGGTATTGATTTTAAAGAAGATGGAAACATTCT
TGGACACAAATTGGAATACAACTATAACTCACACAATGTATACATCATGGCAGACAAACAAA
AGAATGGAATCAAAGTTAACTTCAAAATTAGACACAACATTGAAGATGGAAGCGTTCAACTA
GCAGACCATTATCAACAAAATACTCCAATTGGCGATGGCCCTGTCCTTTTACCAGACAACC
ATTACCTGTCCACACAATCTGCCCTTTCGAAAGATCCCAACGAAAAGAGAGACCACATGGT
CCTTCTTGAGTTTGTAACAGCTGCTGGGATTACACATGGCATGGATGAACTATACAAATAGT
ACCGTTCGTATAGCATACATTATACGAACGGTAGGATGAATTGGTGAAGCGCTGAGAAAAC
ACAAACGCCCCCTCTTTTAAAAGGGGGCGTTTTGAATGTTATTTTGAAAGTGAAACAGGGA
GACTTTCTAATCCTCTTAAAAAGACATTTTTTCTCCATTGAATGTCATCAGGTGCAACCGCAA
GTTCAATATCAGGAAATCTCTTCAAAAGTGCTTTAAATGCAATGTGGCCTTCCAGCCTGGCA
AGAGGCGCTCCTAAGCAGAAATGAATGCCAAAACCAAAAGAAATATGTCTATTAGGCGACC
GATTTATATTTAATATTTCGGGGTTCTCAAAAAAATTCGGGTCGCGATTGGCAGATCCGATG
CCTATAAAAATCATGTCTCCTCTTTTGATCGAATGCCCCTTATATGTAAAGTCTTCGATGGC
CCACCGATTTGCCATCATAACGACAGGTGAGGTGTATCGCAGCAATTCTTCAACCGCTGTA
GCGATCATTTCAGGCTGCGCATGCACGCGTTGATCAGGCGCCTCGTTCCACTGAGCGT

>p14072_pIcBReT-sh-pks1k-GFPmut2

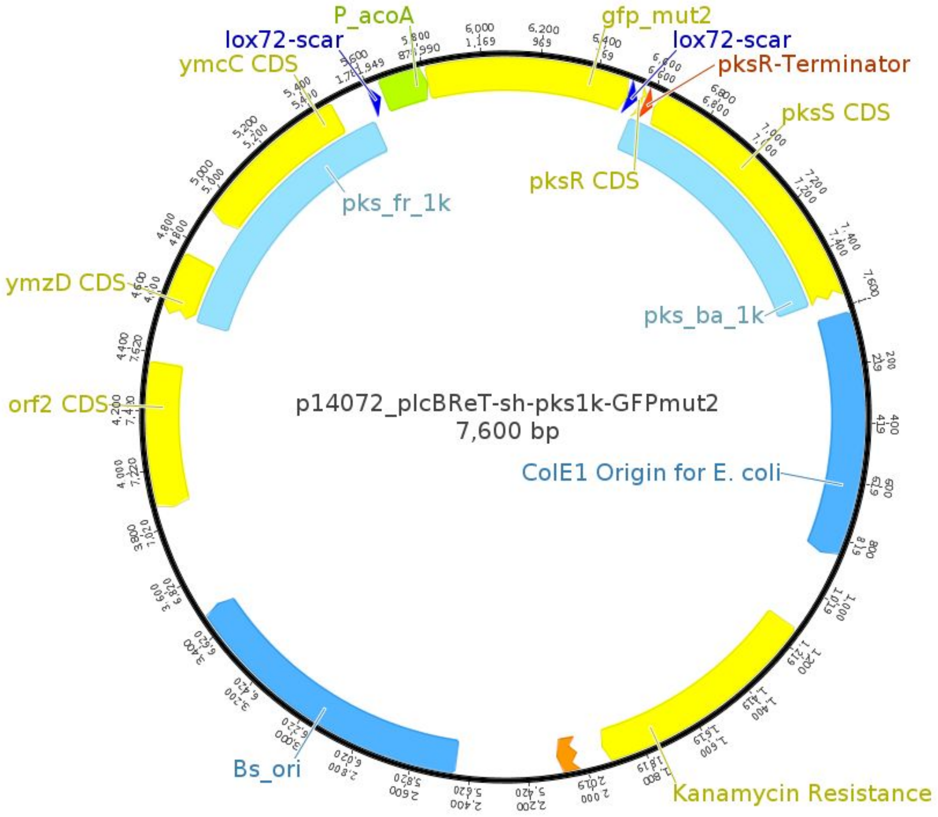

CAGACCCCGTAGAAAAGATCAAAGGATCTTCTTGAGATCCTTTTTTTCTGCGCGTAATCTGC
TGCTTGCAAACAAAAAAACCACCGCTACCAGCGGTGGTTTGTTTGCCGGATCAAGAGCTAC
CAACTCTTTTTCCGAAGGTAACTGGCTTCAGCAGAGCGCAGATACCAAATACTGTTCTTCTA
GTGTAGCCGTAGTTAGGCCACCACTTCAAGAACTCTGTAGCACCGCCTACATACCTCGCTC
TGCTAATCCTGTTACCAGTGGCTGCTGCCAGTGGCGATAAGTCGTGTCTTACCGGGTTGGA
CTCAAGACGATAGTTACCGGATAAGGCGCAGCGGTCGGGCTGAACGGGGGGTTCGTGCA
CACAGCCCAGCTTGGAGCGAACGACCTACACCGAACTGAGATACCTACAGCGTGAGCTAT
GAGAAAGCGCCACGCTTCCCGAAGGGAGAAAGGCGGACAGGTATCCGGTAAGCGGCAGG
GTCGGAACAGGAGAGCGCACGAGGGAGCTTCCAGGGGGAAACGCCTGGTATCTTTATAGT
CCTGTCGGGTTTCGCCACCTCTGACTTGAGCGTCGATTTTTGTGATGCTCGTCAGGGGGG
CGGAGCCTATGGAAAAACGCCAGCAACGCGGCCTTTTTACGGTTCCTGGCCTTTTGCTGG
CCTTTTGCTCACATGTTCTTTCCTGCGTTATCCCCTGATTCTGTGGATAACCGTATTACCGC
CTTTGAGTGAGCTGATACCGCTCGCCGCAGCCGAACGACCGAGCGCAGCGAGTCAGTGA
GCGAGGAAGCGGAAGAGCGCCCAATACGCAAACCGCCTCTCCCCGCGCGTTGGCCGATT
CATTAATGCAGGTTGATCACCTAGAGGATCCCCCAAAGCATAAAAACTTGCATGGACTAAT
GCTTGAAACCCAGGACAATAACCTTATAGCTTGTAAATTCTATCATAATTGTGGTTTCAAAAT
CGGCTCCGTCGATACTATGTTATACGCCAACTTTGAAAACAACTTTGAAAAAGCTGTTTTCT
GGTATTTAAGGTTTTAGAATGCAAGGAACAGTGAATTGGAGTTCGTCTTGTTATAATTAGCT
TCTTGGGGTATCTTTAAATACTGTAGAAAAGAGGAAGGAAATAATAAATGGCTAAAATGAGA
ATATCACCGGAATTGAAAAAACTGATCGAAAAATACCGCTGCGTAAAAGATACGGAAGGAA
TGTCTCCTGCTAAGGTATATAAGCTGGTGGGAGAAAATGAAAACCTATATTTAAAAATGACG
GACAGCCGGTATAAAGGGACCACCTATGATGTGGAACGGGAAAAGGACATGATGCTATGG

CTGGAAGGAAAGCTGCCTGTTCCAAAGGTCCTGCACTTTGAACGGCATGATGGCTGGAGC
AATCTGCTCATGAGTGAGGCCGATGGCGTCCTTTGCTCGGAAGAGTATGAAGATGAACAAA
GCCCTGAAAAGATTATCGAGCTGTATGCGGAGTGCATCAGGCTCTTTCACTCCATCGACAT
ATCGGATTGTCCCTATACGAATAGCTTAGACAGCCGCTTAGCCGAATTGGATTACTTACTGA
ATAACGATCTGGCCGATGTGGATTGCGAAAACTGGGAAGAAGACACTCCATTTAAAGATCC
GCGCGAGCTGTATGATTTTTTAAAGACGGAAAAGCCCGAAGAGGAACTTGTCTTTTCCCAC
GGCGACCTGGGAGACAGCAACATCTTTGTGAAAGATGGCAAAGTAAGTGGCTTTATTGATC
TTGGGAGAAGCGGCAGGGCGGACAAGTGGTATGACATTGCCTTCTGCGTCCGGTCGATCA
GGGAGGATATCGGGGAAGAACAGTATGTCGAGCTATTTTTTGACTTACTGGGGATCAAGCC
TGATTGGGAGAAAATAAAATATTATATTTTACTGGATGAATTGTTTTAGTACCTAGATTTAGA
TGTCTAAAAAGCCTGCAGTCGCGAGGCCTCGACGGATCCCCAAAGCACTTGCATAGGCTA
ATGCCTGGCTTGGTTTGTTGAGACATTACCTGAGAGCCAAAAAACTTTTTATGAATATGAAT
TAAAAAAAAGAACCAACAAAGGCTGAGACAGACTCCAAACGAGTCTGTTTTTTTAAAAAAAA
TATTAGGAGCATTGAATATATATTAGAGAATTAAGAAAGACATGGGAATAAAAATATTTTAAA
TCCAGTAAAAATATGATAAGATTATTTCAGAATATGAAGAACTCTGTTTGTTTTTGATGAAAA
AACAAACAAAAAAAATCCACCTAACGGAATCTCAATTTAACTAACAGCGGCCAAACTGAGAA
GTTAAATTTGAGAAGGGGAAAAGGCGGATTTATACTTGTATTTAACTATCTCCATTTTAACAT
TTTATTAAACCCCATACAAGTGAAAATCCTCTTTTACACTGTTCCTTTAGGTGATCGCGGAG
GGACATTATGAGTGAAGTAAACCTAAAAGGAAATACAGATGAATTAGTGTATTATCGACAGC
AAACCACTGGAAATAAAATCGCCAGGAAGAGAATCAAAAAAGGGAAAGAAGAAGTTTATTA
TGTTGCTGAAACGGAAGAGAAGATATGGACAGAAGAGCAAATAAAAAACTTTTCTTTAGACA
AATTTGGTACGCATATACCTTACATAGAAGGTCATTATACAATCTTAAATAATTACTTCTTTG
ATTTTTGGGGCTATTTTTTAGGTGCTGAAGGAATTGCGCTCTATGCTCACCTAACTCGTTAT
GCATACGGCAGCAAAGACTTTTGCTTTCCTAGTCTACAAACAATCGCTAAAAAAATGGACAA
GACTCCTGTTACAGTTAGAGGCTACTTGAAACTGCTTGAAAGGTACGGTTTTATTTGGAAG
GTAAACGTCCGTAATAAAACCAAGGATAACACAGAGGAATCCCCGATTTTTAAGATTAGAC
GTAAGGTTCCTTTGCTTTCAGAAGAACTTTTAAATGGAAACCCTAATATTGAAATTCCAGAT
GACGAGGAAGCACATGTAAAGAAGGCTTTAAAAAAGGAAAAAGAGGGTCTTCCAAAGGTTT
TGAAAAAAGAGCACGATGAATTTGTTAAAAAAATGATGGATGAGTCAGAAACAATTAATATT
CCAGAGGCCTTACAATATGACACAATGTATGAAGATATACTCAGTAAAGGAGAAATTCGAAA
AGAAATCAAAAAACAAATACCTAATCCTACAACATCTTTTGAGAGTATATCAATGACAACTGA
AGAGGAAAAAGTCGACAGTACTTTAAAAAGCGAAATGCAAAATCGTGTCTCTAAGCCTTCTT
TTGATACCTGGTTTAAAAACACTAAGATCAAAATTGAAAATAAAAATTGTTTATTACTTGTAC
CGAGTGAATTTGCATTTGAATGGATTAAGAAAAGATATTTAGAAACAATTAAAACAGTCCTT
GAAGAAGCTGGATATGTTTTCGAAAAAATCGAACTAAGAAAAGTGCAATAAACTGCTGAAGT
ATTTCAGCAGTTTTTTTTATTTAGAAATAGTGAAAAAAATATAATCAGGGAGGTATCAATATT
TAATGAGTACTGATTTAAATTTATTTAGACTGGAATTAATAATTAACACGTAGACTAATTAAAA
TTTAATGAGGGATAAAGAGGATACAAAAATATTAATTTCAATCCCTATTAAATTTTAACAAGG
GGGGGATTAAAATTTAATTAGAGGTTTATCCACAAGAAAAGACCCTAATAAAATTTTTACTAG
GGTTATAACACTGATTAATTTCTTAATGGGGGAGGGATTAAAATTTAATGACAAAGAAAACA
ATCTTTTAAGAAAAGCTTTTAAAAGATAATAATAAAAAGAGCTTTGCGATTAAGCAAAACTCT
TTACTTTTTCATTGACATTATCAAATTCATCGATTTCAAATTGTTGTTGTATCATAAAGTTAAT
TCTGTTTTGCACAACCTTTTCAGGAATATAAAACACATCTGAGGCTTGTTTTATAAACTCAGG
GTCGCTAAAGTCAATGTAACGTAGCATATGATATGGTATAGCTTCCACCCAAGTTAGCCTTT
CTGCTTCTTCTGAATGTTTTTCATATACTTCCATGGGTATCTCTAAATGATTTTCCTCATGTA
GCAAGGTATGAGCAAAAAGTTTATGGAATTGATAGTTCCTCTCTTTTTCTTCAACTTTTTTAT
CTAAAACAAACACTTTAACATCTGAGTCAATGTAAGCATAAGATGTTTTTCCAGTCATAATTT
CAATCCCAAATCTTTTAGACAGAAATTCTGGACGTAAATCTTTTGGTGAAAGAATTTTTTTAT

GTAGCAATATATCCGATACAGCACCTTCTAAAAGCGTTGGTGAATAGGGCATTTTACCTATC
TCCTCTCATTTTGTGGAATAAAAATAGTCATATTCGTCCATCTACCTATCCTATTATCGAACA
GTTGAACTTTTTAATCAAGGATCAGTCCTTTTTTTCATTATTCTTAAACTGTGCTCTTAACTTT
AACAACTCGCAATCTTATCGGGCTATGCATGCACCTACAGCAAAGCAGGCTAAAATAAATA
AAGCTAATGTTTGATTGGAAGCGTATGTGATGCCAAGTATGGAGAATAAGCCTTTCATCCC
GAAGAAAAAGCCGCCGAAGATGACTGCAAGCGCGATGGCCGCGATAAAAGTAATAGATAC
AATTCCGATTAATTTTGATCTCAGGTTAAAATCTTTAGAGTGCTCGTTCTGTTCTTTTTGGTT
CATGTATGACACTCCCTTCACCTGCTTACTCATACAAACAGCACCCTTGAATCAGCCCAAAA
TATCTATAAGTCTGTTTTCTGACGAAAATAGTATAGCATATTTTAACAAAACCAAAAAAGGCC
CTTTCCACTGGGGAAGGGCCTTTTTCATCATGTTAACTCCTGAGATTCGCCGAAGCTTTCTC
TTTTTTTGGCCAAATAAAATAGCTGGCTGTAATGAGAAAGTCAATGCCTATAATGACAGTCC
ACAGTTTAAGAATGCCGCTCAACGCCTCGGTTCGGGAAGAGTCATTGATAAAATAGATCAT
CCCTGCCAATAGTCCCGCACCAATTAAGTAGGCCAGCACATGTCTTAGCCAGCCTTTCGCA
CCGTGCTTGGCATGGTCCATTCCAAAACGTTTCAGCGGTTTTGTTCCTTTTTTTGTCACATA
ATATTGAAACTTTTCATCTGCCCATTGAATCATTTGTTTCCCATAAGCGATCGATATACCGAT
GTAAACAGCTGCAATCCCATGGGCTGCGGTCGCTGAGGCTCCGCGGTAAAGGTCCACACC
AGTTGCCGCTAACAAGATCAAATCGATAACTGGAGTTAAGGCAAGGAATAGCAGTCCTAAT
GTATGTCGTTTGAATACGTAGCGTACAGCAAGCCCCAAAACAATGACAACCCAAAACGCAA
TTTCACAGAAAACAATCATCCATGCGATACCGTTCAATCCATTCCCCTCTTTTCAATACAGTT
GTATTATTTAAATAATATGTTACCATCTTTGTTTTTGTAACACAACTGTATTATAAAAATTGCC
CTCTCATTTTTTTCTTGTTACGAATTGGATCCTCGAGGCCTACCGTTCGTATAGCATACATTA
TACGAACGGTAGGCCTCTAGAGCATGCTGTAAAACGAGACAAATGAATCAGTTTGAGACAA
AACGAGACACACGTCTCAAACTGTCTCCAAAGTGAAGATGAGAAGACTGATTTTACGGGCT
CAAAAGACTGGCACACTTCTTGCATTTATAATGGTGAACCCTAAATAGAAGGAGGCGCACA
AAATGAGTAAAGGAGAAGAACTTTTCACTGGAGTTGTCCCAATTCTTGTTGAATTAGATGGT
GATGTTAATGGGCACAAATTTTCTGTCAGTGGAGAGGGTGAAGGTGATGCAACATACGGAA
AACTTACCCTTAAATTTATTTGCACTACTGGAAAACTACCTGTTCCATGGCCAACACTTGTC
ACTACTTTCGCGTATGGTCTTCAATGCTTTGCGAGATACCCAGATCATATGAAACAGCATGA
CTTTTTCAAGAGTGCCATGCCCGAAGGTTATGTACAGGAAAGAACTATATTTTTCAAAGATG
ACGGGAACTACAAGACACGTGCTGAAGTCAAGTTTGAAGGTGATACCCTTGTTAATAGAAT
CGAGTTAAAAGGTATTGATTTTAAAGAAGATGGAAACATTCTTGGACACAAATTGGAATACA
ACTATAACTCACACAATGTATACATCATGGCAGACAAACAAAAGAATGGAATCAAAGTTAAC
TTCAAAATTAGACACAACATTGAAGATGGAAGCGTTCAACTAGCAGACCATTATCAACAAAA
TACTCCAATTGGCGATGGCCCTGTCCTTTTACCAGACAACCATTACCTGTCCACACAATCTG
CCCTTTCGAAAGATCCCAACGAAAAGAGAGACCACATGGTCCTTCTTGAGTTTGTAACAGC
TGCTGGGATTACACATGGCATGGATGAACTATACAAATAGTACCGTTCGTATAGCATACATT
ATACGAACGGTAGGATGAATTGGTGAAGCGCTGAGAAAACACAAACGCCCCCTCTTTTAAA
AGGGGGCGTTTTGAATGTTATTTTGAAAGTGAAACAGGGAGACTTTCTAATCCTCTTAAAAA
GACATTTTTTCTCCATTGAATGTCATCAGGTGCAACCGCAAGTTCAATATCAGGAAATCTCT
TCAAAAGTGCTTTAAATGCAATGTGGCCTTCCAGCCTGGCAAGAGGCGCTCCTAAGCAGAA
ATGAATGCCAAAACCAAAAGAAATATGTCTATTAGGCGACCGATTTATATTTAATATTTCGG
GGTTCTCAAAAAAATTCGGGTCGCGATTGGCAGATCCGATGCCTATAAAAATCATGTCTCC
TCTTTTGATCGAATGCCCCTTATATGTAAAGTCTTCGATGGCCCACCGATTTGCCATCATAA
CGACAGGTGAGGTGTATCGCAGCAATTCTTCAACCGCTGTAGCGATCATTTCAGGCTGCTG
CTTGAGCTTCTCACATTCCTTCTTGTGCTGCAGCAATGCGAGGGTGCCTGAGCCGAGTAAG
TTAACAGTTGTTTCAAGGCCGGCTACAACGAGCAAGAACAGCATCGAATAGAGCTCTTTTT
CGCTTAACTTGCTGCCGTTTTCCTCAGCATGCACAAGTTTGCTGATTAAATCGTCTTTTGGC
TTTATTCTTCTGTCATGGATCAGCTTAGCGATATAATCTTTAAATTCACGAAGGGCCTGATTT

GTCAGCTCTCTATTACCTTCAGAGGTATCAACCATCGCATTGGTCCAGATTTGAAACTGTGA
CCGATCTTCTTTTGGGATTCCCATCAATTCAGATATAACAATAAAAGGCAAAGGGGAAGCG
AAGGATTTCATGATATCCGCTTTATTTTCTTTTTCCATTTCATCTAAAAGCTGTTCAGCAATTT
GTTCAATGCTGCCGCGCAGATTTTCAATGGTTCGGGGAGTAAATGCTTGATGAACAAGTGA
TCTCAGGCGGGTATGGTCAGGTGTGTCTTGCATGCACGCGTTGATCAGGCGCCTCGTTCC
ACTGAGCGT

>p14073_pIcBReT-sh-pks-GFPmut2

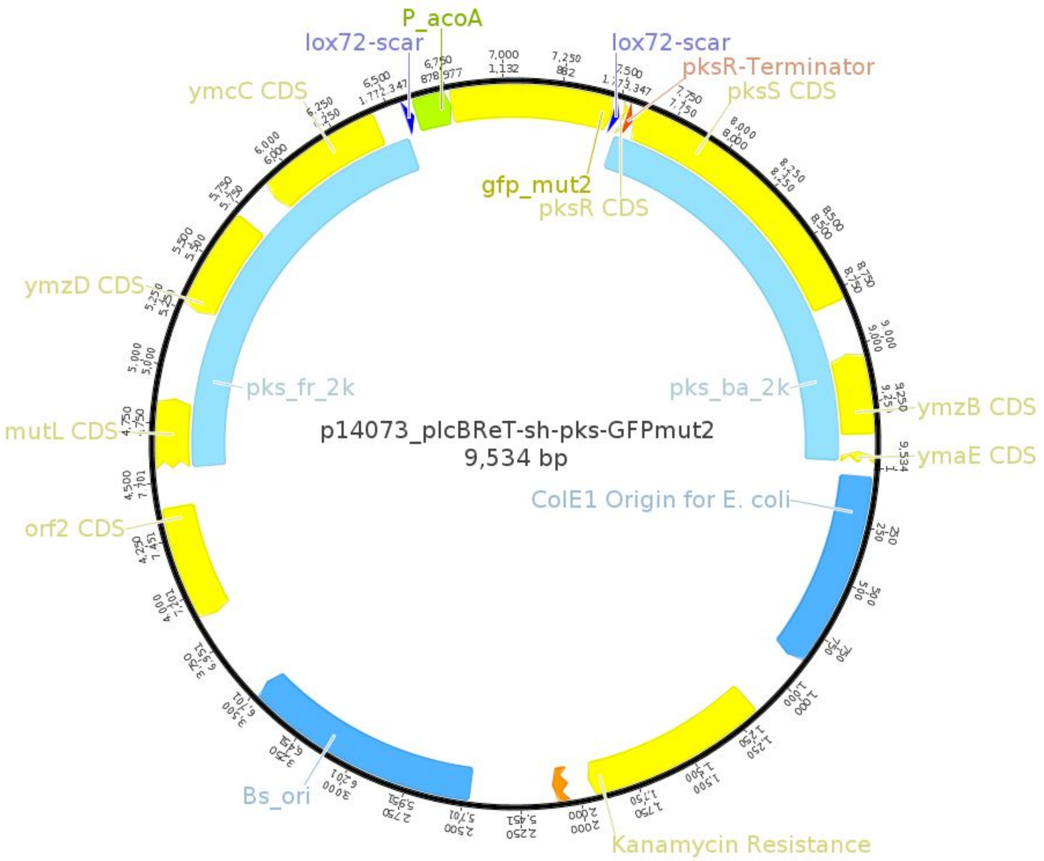

GCCTCGTTCCACTGAGCGTCAGACCCCGTAGAAAAGATCAAAGGATCTTCTTGAGATCCTT
TTTTTCTGCGCGTAATCTGCTGCTTGCAAACAAAAAAACCACCGCTACCAGCGGTGGTTTG
TTTGCCGGATCAAGAGCTACCAACTCTTTTTCCGAAGGTAACTGGCTTCAGCAGAGCGCAG
ATACCAAATACTGTTCTTCTAGTGTAGCCGTAGTTAGGCCACCACTTCAAGAACTCTGTAGC
ACCGCCTACATACCTCGCTCTGCTAATCCTGTTACCAGTGGCTGCTGCCAGTGGCGATAAG
TCGTGTCTTACCGGGTTGGACTCAAGACGATAGTTACCGGATAAGGCGCAGCGGTCGGGC
TGAACGGGGGGTTCGTGCACACAGCCCAGCTTGGAGCGAACGACCTACACCGAACTGAG
ATACCTACAGCGTGAGCTATGAGAAAGCGCCACGCTTCCCGAAGGGAGAAAGGCGGACAG
GTATCCGGTAAGCGGCAGGGTCGGAACAGGAGAGCGCACGAGGGAGCTTCCAGGGGGAA
ACGCCTGGTATCTTTATAGTCCTGTCGGGTTTCGCCACCTCTGACTTGAGCGTCGATTTTT
GTGATGCTCGTCAGGGGGGCGGAGCCTATGGAAAAACGCCAGCAACGCGGCCTTTTTACG
GTTCCTGGCCTTTTGCTGGCCTTTTGCTCACATGTTCTTTCCTGCGTTATCCCCTGATTCTG
TGGATAACCGTATTACCGCCTTTGAGTGAGCTGATACCGCTCGCCGCAGCCGAACGACCG
AGCGCAGCGAGTCAGTGAGCGAGGAAGCGGAAGAGCGCCCAATACGCAAACCGCCTCTC
CCCGCGCGTTGGCCGATTCATTAATGCAGGTTGATCACCTAGAGGATCCCCCAAAGCATAA
AAACTTGCATGGACTAATGCTTGAAACCCAGGACAATAACCTTATAGCTTGTAAATTCTATC
ATAATTGTGGTTTCAAAATCGGCTCCGTCGATACTATGTTATACGCCAACTTTGAAAACAAC
TTTGAAAAAGCTGTTTTCTGGTATTTAAGGTTTTAGAATGCAAGGAACAGTGAATTGGAGTT
CGTCTTGTTATAATTAGCTTCTTGGGGTATCTTTAAATACTGTAGAAAAGAGGAAGGAAATA
ATAAATGGCTAAAATGAGAATATCACCGGAATTGAAAAAACTGATCGAAAAATACCGCTGCG
TAAAAGATACGGAAGGAATGTCTCCTGCTAAGGTATATAAGCTGGTGGGAGAAAATGAAAA
CCTATATTTAAAAATGACGGACAGCCGGTATAAAGGGACCACCTATGATGTGGAACGGGAA

AAGGACATGATGCTATGGCTGGAAGGAAAGCTGCCTGTTCCAAAGGTCCTGCACTTTGAAC
GGCATGATGGCTGGAGCAATCTGCTCATGAGTGAGGCCGATGGCGTCCTTTGCTCGGAAG
AGTATGAAGATGAACAAAGCCCTGAAAAGATTATCGAGCTGTATGCGGAGTGCATCAGGCT
CTTTCACTCCATCGACATATCGGATTGTCCCTATACGAATAGCTTAGACAGCCGCTTAGCC
GAATTGGATTACTTACTGAATAACGATCTGGCCGATGTGGATTGCGAAAACTGGGAAGAAG
ACACTCCATTTAAAGATCCGCGCGAGCTGTATGATTTTTTAAAGACGGAAAAGCCCGAAGA
GGAACTTGTCTTTTCCCACGGCGACCTGGGAGACAGCAACATCTTTGTGAAAGATGGCAAA
GTAAGTGGCTTTATTGATCTTGGGAGAAGCGGCAGGGCGGACAAGTGGTATGACATTGCC
TTCTGCGTCCGGTCGATCAGGGAGGATATCGGGGAAGAACAGTATGTCGAGCTATTTTTG
ACTTACTGGGGATCAAGCCTGATTGGGAGAAAATAAAATATTATATTTTACTGGATGAATTG
TTTTAGTACCTAGATTTAGATGTCTAAAAAGCCTGCAGTCGCGAGGCCTCGACGGATCCCC
AAAGCACTTGCATAGGCTAATGCCTGGCTTGGTTTGTTGAGACATTACCTGAGAGCCAAAA
AACTTTTTATGAATATGAATTAAAAAAAAGAACCAACAAAGGCTGAGACAGACTCCAAACGA
GTCTGTTTTTTTAAAAAAAATATTAGGAGCATTGAATATATATTAGAGAATTAAGAAAGACAT
GGGAATAAAAATATTTTAAATCCAGTAAAAATATGATAAGATTATTTCAGAATATGAAGAACT
CTGTTTGTTTTTGATGAAAAAACAAACAAAAAAAATCCACCTAACGGAATCTCAATTTAACTA
ACAGCGGCCAAACTGAGAAGTTAAATTTGAGAAGGGGAAAAGGCGGATTTATACTTGTATT
TAACTATCTCCATTTTAACATTTTATTAAACCCCATACAAGTGAAAATCCTCTTTTACACTGTT
CCTTTAGGTGATCGCGGAGGGACATTATGAGTGAAGTAAACCTAAAAGGAAATACAGATGA
ATTAGTGTATTATCGACAGCAAACCACTGGAAATAAAATCGCCAGGAAGAGAATCAAAAAA
GGGAAAGAAGAAGTTTATTATGTTGCTGAAACGGAAGAGAAGATATGGACAGAAGAGCAAA
TAAAAAACTTTTCTTTAGACAAATTTGGTACGCATATACCTTACATAGAAGGTCATTATACAA
TCTTAAATAATTACTTCTTTGATTTTTGGGGCTATTTTTTAGGTGCTGAAGGAATTGCGCTCT
ATGCTCACCTAACTCGTTATGCATACGGCAGCAAAGACTTTTGCTTTCCTAGTCTACAAACA
ATCGCTAAAAAAATGGACAAGACTCCTGTTACAGTTAGAGGCTACTTGAAACTGCTTGAAAG
GTACGGTTTTATTTGGAAGGTAAACGTCCGTAATAAAACCAAGGATAACACAGAGGAATCC
CCGATTTTTAAGATTAGACGTAAGGTTCCTTTGCTTTCAGAAGAACTTTTAAATGGAAACCC
TAATATTGAAATTCCAGATGACGAGGAAGCACATGTAAAGAAGGCTTTAAAAAAGGAAAAAG
AGGGTCTTCCAAAGGTTTTGAAAAAAGAGCACGATGAATTTGTTAAAAAAATGATGGATGAG
TCAGAAACAATTAATATTCCAGAGGCCTTACAATATGACACAATGTATGAAGATATACTCAG
TAAAGGAGAAATTCGAAAAGAAATCAAAAAACAAATACCTAATCCTACAACATCTTTTGAGA
GTATATCAATGACAACTGAAGAGGAAAAAGTCGACAGTACTTTAAAAAGCGAAATGCAAAAT
CGTGTCTCTAAGCCTTCTTTTGATACCTGGTTTAAAAACACTAAGATCAAAATTGAAAATAAA
AATTGTTTATTACTTGTACCGAGTGAATTTGCATTTGAATGGATTAAGAAAAGATATTTAGAA
ACAATTAAAACAGTCCTTGAAGAAGCTGGATATGTTTTCGAAAAAATCGAACTAAGAAAAGT
GCAATAAACTGCTGAAGTATTTCAGCAGTTTTTTTTATTTAGAAATAGTGAAAAAAATATAAT
CAGGGAGGTATCAATATTTAATGAGTACTGATTTAAATTTATTTAGACTGGAATTAATAATTA
ACACGTAGACTAATTAAAATTTAATGAGGGATAAAGAGGATACAAAAATATTAATTTCAATCC
CTATTAAATTTTAACAAGGGGGGGATTAAAATTTAATTAGAGGTTTATCCACAAGAAAAGAC
CCTAATAAAATTTTTACTAGGGTTATAACACTGATTAATTTCTTAATGGGGGAGGGATTAAAA
TTTAATGACAAAGAAAACAATCTTTTAAGAAAAGCTTTTAAAAGATAATAATAAAAAGAGCTT
TGCGATTAAGCAAAACTCTTTACTTTTTCATTGACATTATCAAATTCATCGATTTCAAATTGTT
GTTGTATCATAAAGTTAATTCTGTTTTGCACAACCTTTTCAGGAATATAAAACACATCTGAGG
CTTGTTTTATAAACTCAGGGTCGCTAAAGTCAATGTAACGTAGCATATGATATGGTATAGCT
TCCACCCAAGTTAGCCTTTCTGCTTCTTCTGAATGTTTTTCATATACTTCCATGGGTATCTCT
AAATGATTTTCCTCATGTAGCAAGGTATGAGCAAAAAGTTTATGGAATTGATAGTTCCTCTC
TTTTTCTTCAACTTTTTTATCTAAAACAAACACTTTAACATCTGAGTCAATGTAAGCATAAGAT
GTTTTTCCAGTCATAATTTCAATCCCAAATCTTTTAGACAGAAATTCTGGACGTAAATCTTTT

GGTGAAAGAATTTTTTTATGTAGCAATATATCCGATACAGCACCTTCTAAAAGCGTTGGTGA
ATAGGGCATTTTACCTATCTCCTCTCATTTTGTGGAATAAAAATAGTCATATTCGTCCATCTA
CCTATCCTATTATCGAACAGTTGAACTTTTTAATCAAGGATCAGTCCTTTTTTTCATTATTCTT
AAACTGTGCTCTTAACTTTAACAACTCGCAATCTTATCGGGCTATGCATGCTTACATCGTCC
GCTGCCACCCAGCCTGGTTTCCAAAGGGAGAAGAAGCAGAGCTTATAGAAGAAATCATTCA
GCAGGTGCTCGACTCCAAAAATATAGATATTAAAAAACTTCGCGAGGAAGCGGCGATTATG
ATGAGCTGCAAAGGCTCCATCAAAGCAAATCGCCACCTCAGAAACGACGAAATCAAAGCG
CTTCTGGACGACCTCCGAAGCACATCAGACCCATTTACATGCCCGCACGGCCGCCCGATC
ATCATTCACCACTCGACATATGAGATGGAAAAGATGTTCAAACGCGTGATGTAGCGGGGGT
GGTAGGCATTGATCCATTCTCTGAATGGATCAATGCTTTTTTTGTACTCCTTCGAGTGTCAG
ATTATAAGAAACGGTTGTAAACGTGTGAGCAGTGTTAACGCATTATACTTCATGGTTCCTCC
GTATAACAAAATTTCCATTTACAGGCAATTGGGATTAATGTATATTTTGGGTGAGGGGGTGG
AAACATATGGCATGTGGTCCGAAGATCATTATCTTTTTGGATGAACTTGATGATCCAAATGT
GAGAATCATTTTAGAATGCAGAATTTAAATTACAACATTGTGTTTTGCTCCCTGTTCGTGTGA
ACAGGGAGATTTTTTCGTTTATATAACTGGTGATGTCCAAATATAGGGTGTTATGAATTTTCC
ATATCTGCCATCGGCTTTAAGCTGCTTCTCTATTAGGCTGGTTATCAAAAGCAAGTTCAATG
ATAGCCATGAAAGCTGATATCACAACAAGCAAGACATCTGGAATATAAATAGACTGCATAAA
GTAATCTGCAAAACAGATCGTTATAAGATTGCTGATAAAATACAGTATAAAAGCAAAAAGTG
CAGTCTTTTTCAATGAAAGTGATTTTGCCAAAATGATAGAGATAATCTTTGTGAGTGGGTCT
ATGATTAAACCTACAGCAAAGCAGGCTAAAATAAATAAAGCTAATGTTTGATTGGAAGCGTA
TGTGATGCCAAGTATGGAGAATAAGCCTTTCATCCCGAAGAAAAAGCCGCCGAAGATGACT
GCAAGCGCGATGGCCGCGATAAAAGTAATAGATACAATTCCGATTAATTTTGATCTCAGGTT
AAAATCTTTAGAGTGCTCGTTCTGTTCTTTTTGGTTCATGTATGACACTCCCTTCACCTGCTT
ACTCATACAAACAGCACCCTTGAATCAGCCCAAAATATCTATAAGTCTGTTTTCTGACGAAA
ATAGTATAGCATATTTTAACAAAACCAAAAAAGGCCCTTTCCACTGGGGAAGGGCCTTTTTC
ATCATGTTAACTCCTGAGATTCGCCGAAGCTTTCTCTTTTTTTGGCCAAATAAAATAGCTGG
CTGTAATGAGAAAGTCAATGCCTATAATGACAGTCCACAGTTTAAGAATGCCGCTCAACGC
CTCGGTTCGGGAAGAGTCATTGATAAAATAGATCATCCCTGCCAATAGTCCCGCACCAATT
AAGTAGGCCAGCACATGTCTTAGCCAGCCTTTCGCACCGTGCTTGGCATGGTCCATTCCAA
AACGTTTCAGCGGTTTTGTTCCTTTTTTTGTCACATAATATTGAAACTTTTCATCTGCCCATT
GAATCATTTGTTTCCCATAAGCGATCGATATACCGATGTAAACAGCTGCAATCCCATGGGCT
GCGGTCGCTGAGGCTCCGCGGTAAAGGTCCACACCAGTTGCCGCTAACAAGATCAAATCG
ATAACTGGAGTTAAGGCAAGGAATAGCAGTCCTAATGTATGTCGTTTGAATACGTAGCGTA
CAGCAAGCCCCAAAACAATGACAACCCAAAACGCAATTTCACAGAAAACAATCATCCATGC
GATACCGTTCAATCCATTCCCCTCTTTTCAATACAGTTGTATTATTTAAATAATATGTTACCAT
CTTTGTTTTTGTAACACAACTGTATTATAAAAATTGCCCTCTCATTTTTTTCTTGTTACGAATT
GGATCCTCGAGGCCTACCGTTCGTATAGCATACATTATACGAACGGTAGGCCTCTAGAGCA
TGCTGTAAAACGAGACAAATGAATCAGTTTGAGACAAAACGAGACACACGTCTCAAACTGT
CTCCAAAGTGAAGATGAGAAGACTGATTTTACGGGCTCAAAAGACTGGCACACTTCTTGCA
TTTATAATGGTGAACCCTAAATAGAAGGAGGCGCACAAAATGAGTAAAGGAGAAGAACTTT
TCACTGGAGTTGTCCCAATTCTTGTTGAATTAGATGGTGATGTTAATGGGCACAAATTTTCT
GTCAGTGGAGAGGGTGAAGGTGATGCAACATACGGAAAACTTACCCTTAAATTTATTTGCA
CTACTGGAAAACTACCTGTTCCATGGCCAACACTTGTCACTACTTTCGCGTATGGTCTTCAA
TGCTTTGCGAGATACCCAGATCATATGAAACAGCATGACTTTTTCAAGAGTGCCATGCCCG
AAGGTTATGTACAGGAAAGAACTATATTTTTCAAAGATGACGGGAACTACAAGACACGTGCT
GAAGTCAAGTTTGAAGGTGATACCCTTGTTAATAGAATCGAGTTAAAAGGTATTGATTTTAA
AGAAGATGGAAACATTCTTGGACACAAATTGGAATACAACTATAACTCACACAATGTATACA
TCATGGCAGACAAACAAAAGAATGGAATCAAAGTTAACTTCAAAATTAGACACAACATTGAA

GATGGAAGCGTTCAACTAGCAGACCATTATCAACAAAATACTCCAATTGGCGATGGCCCTG
TCCTTTTACCAGACAACCATTACCTGTCCACACAATCTGCCCTTTCGAAAGATCCCAACGAA
AAGAGAGACCACATGGTCCTTCTTGAGTTTGTAACAGCTGCTGGGATTACACATGGCATGG
ATGAACTATACAAATAGTACCGTTCGTATAGCATACATTATACGAACGGTAGGATGAATTGG
TGAAGCGCTGAGAAAACACAAACGCCCCCTCTTTTAAAAGGGGGCGTTTTGAATGTTATTTT
GAAAGTGAAACAGGGAGACTTTCTAATCCTCTTAAAAAGACATTTTTTCTCCATTGAATGTC
ATCAGGTGCAACCGCAAGTTCAATATCAGGAAATCTCTTCAAAAGTGCTTTAAATGCAATGT
GGCCTTCCAGCCTGGCAAGAGGCGCTCCTAAGCAGAAATGAATGCCAAAACCAAAAGAAA
TATGTCTATTAGGCGACCGATTTATATTTAATATTTCGGGGTTCTCAAAAAAATTCGGGTCG
CGATTGGCAGATCCGATGCCTATAAAAATCATGTCTCCTCTTTTGATCGAATGCCCCTTATA
TGTAAAGTCTTCGATGGCCCACCGATTTGCCATCATAACGACAGGTGAGGTGTATCGCAGC
AATTCTTCAACCGCTGTAGCGATCATTTCAGGCTGCTGCTTGAGCTTCTCACATTCCTTCTT
GTGCTGCAGCAATGCGAGGGTGCCTGAGCCGAGTAAGTTAACAGTTGTTTCAAGGCCGGC
TACAACGAGCAAGAACAGCATCGAATAGAGCTCTTTTTCGCTTAACTTGCTGCCGTTTTCCT
CAGCATGCACAAGTTTGCTGATTAAATCGTCTTTTGGCTTTATTCTTCTGTCATGGATCAGC
TTAGCGATATAATCTTTAAATTCACGAAGGGCCTGATTTGTCAGCTCTCTATTACCTTCAGA
GGTATCAACCATCGCATTGGTCCAGATTTGAAACTGTGACCGATCTTCTTTTGGGATTCCCA
TCAATTCAGATATAACAATAAAAGGCAAAGGGGAAGCGAAGGATTTCATGATATCCGCTTTA
TTTTCTTTTTCCATTTCATCTAAAAGCTGTTCAGCAATTTGTTCAATGCTGCCGCGCAGATTT
TCAATGGTTCGGGGAGTAAATGCTTGATGAACAAGTGATCTCAGGCGGGTATGGTCAGGT
GTGTCTTTTGCCAGCATATGATCGGATACAAAATCGATATCTTCACTAACGTTGAGCATTTT
GATTTGTTCTTGGTTCATCACATTTTTTACGTCTCTTGTAATTCGATTGTCTTTTAAAAAGGC
CATACAATCATCGTATCGGGTAATTAACCAGGCCGGATATGTGGCTCCGAACCGTTTTAATT
CAAATCGGTGAATGGGCTCTTCCTCTCTAAATCGTCCTAAAACTGAAAAAGGATTGTGATGA
AACTCTTTACCATGCGGATGAAACATCAATTTTTCCATTTGCATTCTCCTCGCCTAATAGGG
TAAATAGATGAATCAAATTGCTGAATTAGTTTACAAAAAACAGAATGATTTGAAATGTAATCC
TGTCTCTAAAACTATATATCTATCTTAGGCGTCATTCAATAGGGAGAAGAACGAAAAAAGTG
AAAAAACGGCTCGATATAAAGCAGCGCCTTTGAACGAAAGCTCAAAGGCGCTACGCTGTAT
TATTTTGATGAAAGTGGCTGTCAGCTGTGCTGGATATCAATTGTATATACTGCACGATCTGT
TACGACCTTCAATCCTTCGTTTTCTTGGTGAATATGAAGCTCACCTAATAAAGGAATCTCAT
AGGCATTTTGCGGAAGCGGTACGTTTCCGTCCTCAGTGAATGTTGTTCCTTCGCCTTCTAA
AACAAGCTCTTCTTTCGCAACATAATCATCAGGATGATTCGTACTGCGTATAGCCACTTTTT
GAAGGTGGAGAACAATCTGATCCAAGTCTGATATCTCTTCATCATGGGTCAATTCACCTTTT
CTAATATCAAGCGTCTGGCCGACTTTTGATTCCA

GCCATTGAGAAAGCTGTGTATTCGTGTGTGCCATCAATATTCCTCCTTTTGATCTACACGAT
ACTATTCCCAATTGCTGCATCTTTTACACGGGAAAGAGCCGCCCCACTCTTTATGTATGAAA
CAGTTCGCTGTTGAGTGCCTTGATATAGCGTACGCATGCACGCGTTGATCAGGC

>p14094_pMSE3-acoA-amyE-mPLC

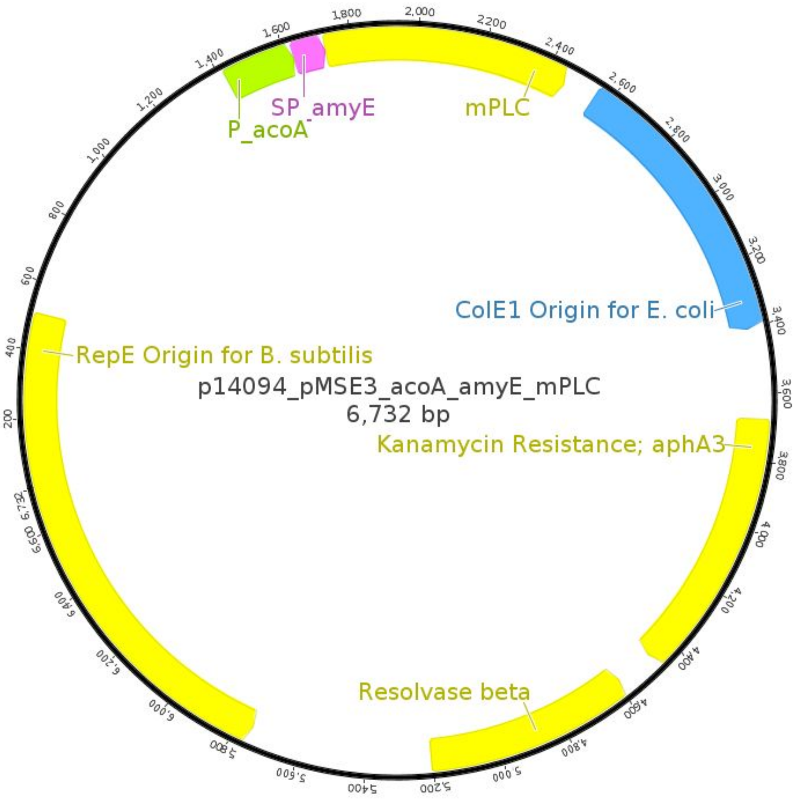

ATTATTTTGGCTGCTTTGACAGATTTAAATTCTGATTTTGAAGTCACATAGACTGGCGTTTCT
AAAACAAAATATGCTTGATAACCTTTATCAGATTTGATAATCATAGTAGGCATAAAACCTAAA
TCAATAGCGGTTGTTAAAATATCGCTTGCTGAAATAGTTTCTTTTGCCGTGTGAATATCAAAA
TCAATAAAGAAGGTATTGATTTGTCTTAAATTGTTTTCAGAATGTCCTTTCGTGTATGAACGG
TTTTCGTCTGCATACGTTCCATAACGATAAACGTTGGGTGTCCAATGTGTAAATGTATCTTG
ATTTTCTTGAATCGCTTCCTCGGAAGTCAGAACAACACCACGACCGCCAATCATGCTTGATT
TTGAGCGATACGCAAAAATAGCCCCTTTGCTTTTACCTGGCTTGGTAGTGATTGAGCGAATT
TTACTATTTTTAAATTTGTACTTTAACAAGCCGTCATGAAGCACAGTTTCTACAACAAAAGGG
ATATTCATTCAGCTGTTCTCCTTTCCTATAAATCCTATAAAATAGGTTGTTTAATTAACTTGGT
TTGCTTTTTCATTCAACTGTTTCAATATTGCATGTTTTGAAAAAGATTTTTTTCCTTTATAAGT
CAATTTTTTTCCACTAATCGAATAAATTATTTTGTTATTTTCTATTAACTTATATATATAATCTT
CCCCCTCCGAAGAAAAATACTTATCTGATTTTGTTTCTAAGTAGATATTTCTCTTTTCTAACT
CTTTCTTAAACGTTTCTAGTGTATAGATATTTGCTAATTTTCTTATCTCCAATAAACTATTTTT
TATATAAGTTTTACATTCATCATGATTCATACAAACTCCACCTTCTATAAATGAATACAAAAAA
AGCAATCAAACGATTTCCGATTGATTGCTTAACAATTCTTAAATTCAGTAGCTTAGATACTTG
AAAACTCTCTGATTTCCCTATATAATGATAGTACGGTTATATACCGTCTTCAAACAAAGTTAA
TTAAATAACTTCTTACGAGGGAAGAGTTCATCTGACTAACTGATAAGCGTTGGTTTGGCAAT
CTTATCGGGCTATGCATTTATAAAATGTCGTCAAACATTTTATAAATGTGTCATGGCTCTTTT
TTCGTTTCTATTCAGTTCGTTGTTTCGTTATATCTAGTATACCGCTTTTAAAAAAAATAAGCA
ACGATTTCGTGCATTATTCACACGAAGTCATTGCTTTTTTCTTCTTCCATTTCTAAATCCAAT
GTTACTTGTTCTGATTCTGTTTCTGGTTCTGGTTCTGTTGGCTCACTCGAGGGTACCGGGG
GGCGCCAGGCCTGGCGACCCGAGGATATCTCTAGATCAGTCAAACGATGCAGAGGAACTA

GGAAGTTAAAAAGATTTCCAAGGAAATAAATACGTCGATCATTGTCAAAGGCCGGGTGATA
TCCGGTCTTTTTTTTGCATGCTGTAAAACGAGACAAATGAATCAGTTTGAGACAAAACGAGA
CACACGTCTCAAACTGTCTCCAAAGTGAAGATGAGAAGACTGATTTTACGGGCTCAAAAGA
CTGGCACACTTCTTGCATTTATAATGGTGAACCCTAAATAGAAGGAGGCGCACAAAATGTTT
GCAAAACGATTCAAAACCTCTTTACTGCCGTTATTCGCTGGATTTTTATTGCTGTTTCATTTG
GTTCTGGCAGGACCGGCGGCTGCGAGTGCTTGGTCTGCTGAAGATAAACATAAAGAAGGC
GTAAATTCTCATTTATGGATTGTAAACCGTGCGATTGATATTATGTCTCGCAATACAACACTT
GTAAAACAAGATCGAGTTGCACAATTAAATGAATGGCGTACAGAGTTAGAGAACGGTATTTA
TGCTGCTGACTATGAAAATCCTTATTATGATAATAGCACATTTGCTTCACATTTCTATGATCC
AGACAATGGAAAAACATATATTCCATTTGCAAAGCAGGCAAAAGAAACTGGAGCTAAATATT
TTAAATTAGCGGGTGAATCATACAAAAATAAAGATATGAAACAAGCATTCTTCTATTTAGGGT
TATCTCTTCATTATTTAGGAGATGTAAATCAACCGATGCATGCGGCAAACTTTACAAATCTTT
CGTATCCACAAGGATTCCATTCTAAATATGAAAACTTTGTAGATACGATAAAAGATAATTATA
AAGTAACGGATGGAAATGGATATTGGAATTGGAAAGGTACAAATCCAGAAGATTGGATCCA
TGGAGCGGCAGTAGTGGCGAAACAAGATTACTCTGGAATTGTAAATGATAATACGAAAGAT
TGGTTCGTAAAAGCAGCTGTGTCACAAGAATATGCAGATAAATGGCGTGCTGAAGTTACAC
CGATGACAGGTAAGCGATTAATGGATGCACAACGTGTTACTGCTGGATACATTCAGCTTTG
GTTTGATACGTACGGAGATCGTTAATCTAGAGAATTCTCGCGATACGTACCCGGGGAATCG
CGGCCGCACGCGTTGATCAGGCGCCTCGTTCCACTGAGCGTCAGACCCCGTAGAAAAGAT
CAAAGGATCTTCTTGAGATCCTTTTTTTCTGCGCGTAATCTGCTGCTTGCAAACAAAAAAAC
CACCGCTACCAGCGGTGGTTTGTTTGCCGGATCAAGAGCTACCAACTCTTTTTCCGAAGGT
AACTGGCTTCAGCAGAGCGCAGATACCAAATACTGTTCTTCTAGTGTAGCCGTAGTTAGGC
CACCACTTCAAGAACTCTGTAGCACCGCCTACATACCTCGCTCTGCTAATCCTGTTACCAG
TGGCTGCTGCCAGTGGCGATAAGTCGTGTCTTACCGGGTTGGACTCAAGACGATAGTTAC
CGGATAAGGCGCAGCGGTCGGGCTGAACGGGGGGTTCGTGCACACAGCCCAGCTTGGAG
CGAACGACCTACACCGAACTGAGATACCTACAGCGTGAGCTATGAGAAAGCGCCACGCTT
CCCGAAGGGAGAAAGGCGGACAGGTATCCGGTAAGCGGCAGGGTCGGAACAGGAGAGC
GCACGAGGGAGCTTCCAGGGGGAAACGCCTGGTATCTTTATAGTCCTGTCGGGTTTCGCC
ACCTCTGACTTGAGCGTCGATTTTTGTGATGCTCGTCAGGGGGGCGGAGCCTATGGAAAA
ACGCCAGCAACGCGGCCTTTTTACGGTTCCTGGCCTTTTGCTGGCCTTTTGCTCACATGTT
CTTTCCTGCGTTATCCCCTGATTCTGTGGATAACCGTATTACCGCCTTTGAGTGAGCTGATA
CCGCTCGCCGCAGCCGAACGACCGAGCGCAGCGAGTCAGTGAGCGAGGAAGCGGAAGA
GCGCCCAATACGCAAACCGCCTCTCCCCGCGCGTTGGCCGATTCATTAATGCAGGTTGAT
CACCTAGAGGATCCCCCAAAGCATAAAAACTTGCATGGACTAATGCTTGAAACCCAGGACA
ATAACCTTATAGCTTGTAAATTCTATCATAATTGTGGTTTCAAAATCGGCTCCGTCGATACTA
TGTTATACGCCAACTTTGAAAACAACTTTGAAAAAGCTGTTTTCTGGTATTTAAGGTTTTAGA
ATGCAAGGAACAGTGAATTGGAGTTCGTCTTGTTATAATTAGCTTCTTGGGGTATCTTTAAA
TACTGTAGAAAAGAGGAAGGAAATAATAAATGGCTAAAATGAGAATATCACCGGAATTGAAA
AAACTGATCGAAAAATACCGCTGCGTAAAAGATACGGAAGGAATGTCTCCTGCTAAGGTAT
ATAAGCTGGTGGGAGAAAATGAAAACCTATATTTAAAAATGACGGACAGCCGGTATAAAGG
GACCACCTATGATGTGGAACGGGAAAAGGACATGATGCTATGGCTGGAAGGAAAGCTGCC
TGTTCCAAAGGTCCTGCACTTTGAACGGCATGATGGCTGGAGCAATCTGCTCATGAGTGAG
GCCGATGGCGTCCTTTGCTCGGAAGAGTATGAAGATGAACAAAGCCCTGAAAAGATTATCG
AGCTGTATGCGGAGTGCATCAGGCTCTTTCACTCCATCGACATATCGGATTGTCCCTATAC
GAATAGCTTAGACAGCCGCTTAGCCGAATTGGATTACTTACTGAATAACGATCTGGCCGAT
GTGGATTGCGAAAACTGGGAAGAAGACACTCCATTTAAAGATCCGCGCGAGCTGTATGATT
TTTTAAAGACGGAAAAGCCCGAAGAGGAACTTGTCTTTTCCCACGGCGACCTGGGAGACA
GCAACATCTTTGTGAAAGATGGCAAAGTAAGTGGCTTTATTGATCTTGGGAGAAGCGGCAG

GGCGGACAAGTGGTATGACATTGCCTTCTGCGTCCGGTCGATCAGGGAGGATATCGGGGA
AGAACAGTATGTCGAGCTATTTTTTGACTTACTGGGGATCAAGCCTGATTGGGAGAAAATAA
AATATTATATTTTACTGGATGAATTGTTTTAGTACCTAGATTTAGATGTCTAAAAAGCCTGCA
GTCGCGAGGCCTCGACGGATCCCCAAAGCACTTGCATAGGCTAATGCCTGGCTTGGTTTT
TCAGCTAAAATAACCGTACTCATTAACTATCCCTCTTTTCATTGTTTTTTCTTTGATCGACTG
TCACGTTGTATCTTGATCGATACCTTCTAAACGTTCGGCGATTAATTCCAGTTTGTTCTTCAA
CTTCTTTATCGGATAAACCGTTCAAAAACAAATCGAAAGCGTGTTGTAAACGTGGATCATTT
TCTTTGAATTTCAGTTGTCGCCCTTTGAATTTTCCTTTTTTCTTAGCAATTTCAATTCCTTGG
GCTTGGCGTTCTTTAATTCGTTTGCGTTCAGATTCCGCTTGGTACTTATACAATTCAATCACT
AAATTATTAATCAGCCGTCTTAAATTTTCATCTTCAATACCATTCATTGAGGGTAAATTTAAG
ACTTCCAGGGTTGCCCCCTTAATTTGAATTTGATTCATCAATTCTGTTAATTCTTTATTATTTC
GTCCTAATCGATCTAATTCAGTAACAACAACTATATCCCCTTCACGAATATAGTTAAGCATA
GCTTGTAATTGTGGGCGTTCGACCGATTGACCGCTTGCTTTGTCTGAAAAGACCTTAGAAA
CGCCCTCTAACGCTTTCAGTTGTCTATCTAAGTTCTGTTCTTTGCTACTGACACGTGCATAC
CCTACTTTAGTCATGTTAAAAAACACCTCCCTTGTTAAGAAAAAAATAAATTGGATATGAATT
GGGAATTCTATTTAATCACTTTGACTAGCAAATACTAACAACAAGACACACACACCAAAAAT
CAAAAATTCACTACTTTTAGTTAAAAACCACGTAACCACAAGAACTAATCCAATCCATGTAAT
CGGGTTCTTCAAATATTTCTCCAAGATTTTCCTCCTCTAATATGCTCAACTTAAATGACCTAT
TCAATAAATCTATTATGCTGCTAAATAGTTTATAGGACAAATAAGTATACTCTAATGACCTAT
AAAAGATAGAAAATTAAAAAATCAAGTGTTCGCTTCGCTCTCACTGCCCCTCGACGTTTTAG
TAGCCTTTCCCTCACTTCGTTCAGTCCAAGCCAACTAAAAGTTTTCGGGCTACTCTCTCCTT
CTCCCCCTAATAATTAATTAAAATCTTACTCTGTATATTTCTGCTAATCATTCGCTAAACAGC
AAAGAAAAAACAAACACGTATCATAGATATAAATGTAATGGCATAGTGCGGGTTTTATTTTC
AGCCTGTATCATAGCTAAACAAATCGAGTTGTGTGTCCGTTTTAGGGCGTTCTGCTAGCTT
GTTTAAAGTCTCTTGAATGAATGTATGCTCTAAGTCAAAAGAATTTGTCAGCGCCTTTATATA
GCTTTCTTTTTCTTCTTTTTTTACTTTAATGATCGATAGCAACAATGATTTAACACTAGCAAGT
TGAATGCCACCATTTCTTCCTGGTTTAATCTTAAAGAAAATTTCCTGATTCGCCTTCAGTACC
TTCAGCAATTTATCTAATGTCCGTTCAGGAATGCCTAGCACTTCTCTAATCTCTTTTTTGGTC
GTCACTAAATAAGGCTTGTATACATCGCTTTTTTCGCTAATATAAGCCATTAAATCTTCTTTC
CATTCTGACAAATGAACACGTTGACGTTCGCTTCTTTTTTTCTTGAATTTAAACCACCCTTGA
CGGACAAATAAATCTTTACTGGTTAAATCACTTGATACCCAAGCTTTGCAAAGAATGGTAAT
GTATTCCCTATTAGCCCCTTGATAGTTTTCTGAATAGGCACTTCTAACAATTTTGATTACTTC
TTTTTCTTCTAAGGGTTGATCTAATCGATTATTAAACTCAAACATATTATATTCGCACGTTTC
GATTGAATAGCCTGAACTAAAGTAGGCTAAAGAGAGGGTAAACATGACGTTATTACGCCCT
ATTAAACCCTTTTCTCCTGAAAATTTCGTTTCGTGCAATAAGAGATTAAACCAGGGTTCATCT
ACTTGTTTTTTGCCTTCTGTACCGCTTAAAACCGTTAGACTTGAACGAGTAAAGCCCTTATT
ATCTGTTTGTTTGAAAGACCAATCTTGCCATTCTTTGAAAGAATAACGGTAATTAGGATCAAA
AAATTCTACATTGTCCGTTCTTGGTATGCGAGCAATACCAAAATGATTACACGTTAGATCAA
CTGGCAAAGACTTTCCAAAATATTCTCGGATATTTTGCGAA

>p14096_plc-pks2k-mPLC

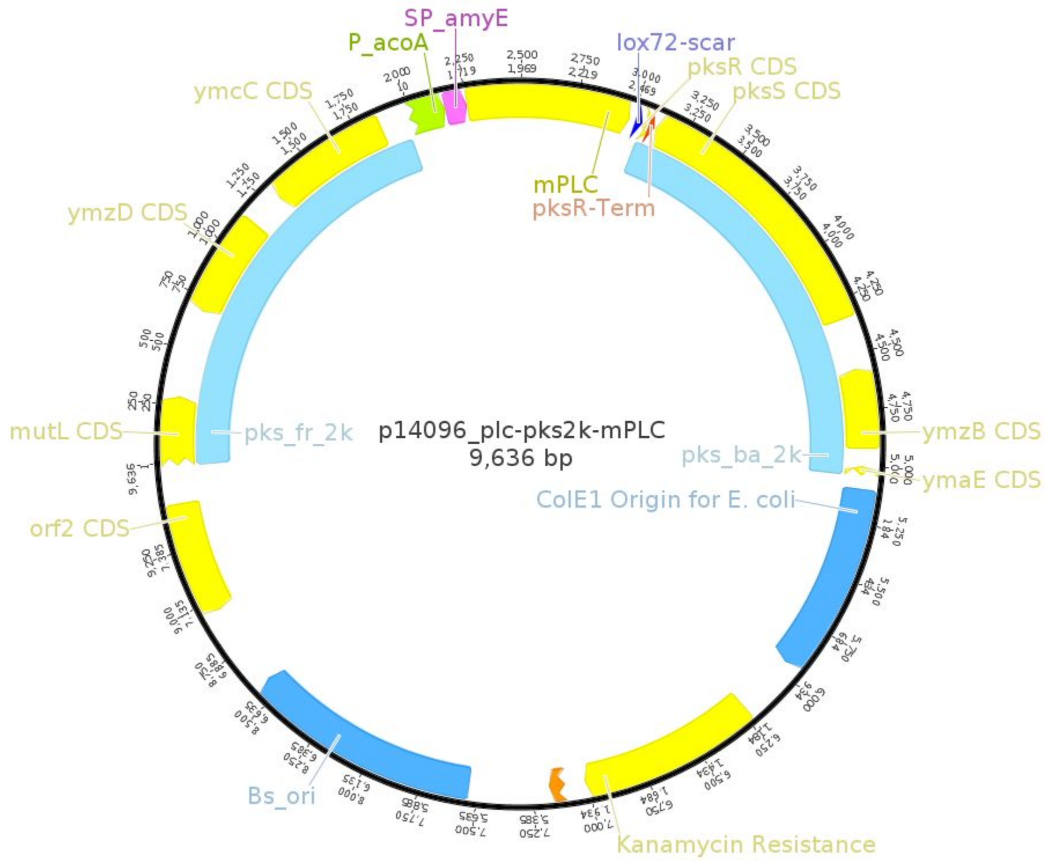

CTGCCACCCAGCCTGGTTTCCAAAGGGAGAAGAAGCAGAGCTTATAGAAGAAATCATTCAG
CAGGTGCTCGACTCCAAAAATATAGATATTAAAAAACTTCGCGAGGAAGCGGCGATTATGA
TGAGCTGCAAAGGCTCCATCAAAGCAAATCGCCACCTCAGAAACGACGAAATCAAAGCGCT
TCTGGACGACCTCCGAAGCACATCAGACCCATTTACATGCCCGCACGGCCGCCCGATCAT
CATTCACCACTCGACATATGAGATGGAAAAGATGTTCAAACGCGTGATGTAGCGGGGGTG
GTAGGCATTGATCCATTCTCTGAATGGATCAATGCTTTTTTTGTACTCCTTCGAGTGTCAGA
TTATAAGAAACGGTTGTAAACGTGTGAGCAGTGTTAACGCATTATACTTCATGGTTCCTCCG
TATAACAAAATTTCCATTTACAGGCAATTGGGATTAATGTATATTTTGGGTGAGGGGGTGGA
AACATATGGCATGTGGTCCGAAGATCATTATCTTTTTGGATGAACTTGATGATCCAAATGTG
AGAATCATTTTAGAATGCAGAATTTAAATTACAACATTGTGTTTTGCTCCCTGTTCGTGTGAA
CAGGGAGATTTTTTCGTTTATATAACTGGTGATGTCCAAATATAGGGTGTTATGAATTTTCCA
TATCTGCCATCGGCTTTAAGCTGCTTCTCTATTAGGCTGGTTATCAAAAGCAAGTTCAATGA
TAGCCATGAAAGCTGATATCACAACAAGCAAGACATCTGGAATATAAATAGACTGCATAAAG
TAATCTGCAAAACAGATCGTTATAAGATTGCTGATAAAATACAGTATAAAAGCAAAAAGTGC
AGTCTTTTTCAATGAAAGTGATTTTGCCAAAATGATAGAGATAATCTTTGTGAGTGGGTCTAT
GATTAAACCTACAGCAAAGCAGGCTAAAATAAATAAAGCTAATGTTTGATTGGAAGCGTATG
TGATGCCAAGTATGGAGAATAAGCCTTTCATCCCGAAGAAAAAGCCGCCGAAGATGACTGC
AAGCGCGATGGCCGCGATAAAAGTAATAGATACAATTCCGATTAATTTTGATCTCAGGTTAA
AATCTTTAGAGTGCTCGTTCTGTTCTTTTTGGTTCATGTATGACACTCCCTTCACCTGCTTAC
TCATACAAACAGCACCCTTGAATCAGCCCAAAATATCTATAAGTCTGTTTTCTGACGAAAAT
AGTATAGCATATTTTAACAAAACCAAAAAAGGCCCTTTCCACTGGGGAAGGGCCTTTTTCAT
CATGTTAACTCCTGAGATTCGCCGAAGCTTTCTCTTTTTTTGGCCAAATAAAATAGCTGGCT

GTAATGAGAAAGTCAATGCCTATAATGACAGTCCACAGTTTAAGAATGCCGCTCAACGCCT
CGGTTCGGGAAGAGTCATTGATAAAATAGATCATCCCTGCCAATAGTCCCGCACCAATTAA
GTAGGCCAGCACATGTCTTAGCCAGCCTTTCGCACCGTGCTTGGCATGGTCCATTCCAAAA
CGTTTCAGCGGTTTTGTTCCTTTTTTTGTCACATAATATTGAAACTTTTCATCTGCCCATTGA
ATCATTTGTTTCCCATAAGCGATCGATATACCGATGTAAACAGCTGCAATCCCATGGGCTG
CGGTCGCTGAGGCTCCGCGGTAAAGGTCCACACCAGTTGCCGCTAACAAGATCAAATCGA
TAACTGGAGTTAAGGCAAGGAATAGCAGTCCTAATGTATGTCGTTTGAATACGTAGCGTAC
AGCAAGCCCCAAAACAATGACAACCCAAAACGCAATTTCACAGAAAACAATCATCCATGCG
ATACCGTTCAATCCATTCCCCTCTTTTCAATACAGTTGTATTATTTAAATAATATGTTACCATC
TTTGTTTTTGTAACACAACTGTATTATAAAAATTGCCCTCTCATTTTTTTCTTGTTACGAATTG
GATCCTCGAGGCCGCATGCTGTAAAACGAGACAAATGAATCAGTTTGAGACAAAACGAGAC
ACACGTCTCAAACTGTCTCCAAAGTGAAGATGAGAAGACTGATTTTACGGGCTCAAAAGAC
TGGCACACTTCTTGCATTTATAATGGTGAACCCTAAATAGAAGGAGGCGCACAAAATGTTTG
CAAAACGATTCAAAACCTCTTTACTGCCGTTATTCGCTGGATTTTTATTGCTGTTTCATTTGG
TTCTGGCAGGACCGGCGGCTGCGAGTGCTTGGTCTGCTGAAGATAAACATAAAGAAGGCG
TAAATTCTCATTTATGGATTGTAAACCGTGCGATTGATATTATGTCTCGCAATACAACACTTG
TAAAACAAGATCGAGTTGCACAATTAAATGAATGGCGTACAGAGTTAGAGAACGGTATTTAT
GCTGCTGACTATGAAAATCCTTATTATGATAATAGCACATTTGCTTCACATTTCTATGATCCA
GACAATGGAAAAACATATATTCCATTTGCAAAGCAGGCAAAAGAAACTGGAGCTAAATATTT
TAAATTAGCGGGTGAATCATACAAAAATAAAGATATGAAACAAGCATTCTTCTATTTAGGGTT
ATCTCTTCATTATTTAGGAGATGTAAATCAACCGATGCATGCGGCAAACTTTACAAATCTTTC
GTATCCACAAGGATTCCATTCTAAATATGAAAACTTTGTAGATACGATAAAAGATAATTATAA
AGTAACGGATGGAAATGGATATTGGAATTGGAAAGGTACAAATCCAGAAGATTGGATCCAT
GGAGCGGCAGTAGTGGCGAAACAAGATTACTCTGGAATTGTAAATGATAATACGAAAGATT
GGTTCGTAAAAGCAGCTGTGTCACAAGAATATGCAGATAAATGGCGTGCTGAAGTTACACC
GATGACAGGTAAGCGATTAATGGATGCACAACGTGTTACTGCTGGATACATTCAGCTTTGG
TTTGATACGTACGGAGATCGTTAATCTAGAGAATTCTCGCGATACGTACCTACCGTTCGTAT
AGCATACATTATACGAACGGTAGGATGAATTGGTGAAGCGCTGAGAAAACACAAACGCCCC
CTCTTTTAAAAGGGGGCGTTTTGAATGTTATTTTGAAAGTGAAACAGGGAGACTTTCTAATC
CTCTTAAAAAGACATTTTTTCTCCATTGAATGTCATCAGGTGCAACCGCAAGTTCAATATCA
GGAAATCTCTTCAAAAGTGCTTTAAATGCAATGTGGCCTTCCAGCCTGGCAAGAGGCGCTC
CTAAGCAGAAATGAATGCCAAAACCAAAAGAAATATGTCTATTAGGCGACCGATTTATATTT
AATATTTCGGGGTTCTCAAAAAAATTCGGGTCGCGATTGGCAGATCCGATGCCTATAAAAAT
CATGTCTCCTCTTTTGATCGAATGCCCCTTATATGTAAAGTCTTCGATGGCCCACCGATTTG
CCATCATAACGACAGGTGAGGTGTATCGCAGCAATTCTTCAACCGCTGTAGCGATCATTTC
AGGCTGCTGCTTGAGCTTCTCACATTCCTTCTTGTGCTGCAGCAATGCGAGGGTGCCTGAG
CCGAGTAAGTTAACAGTTGTTTCAAGGCCGGCTACAACGAGCAAGAACAGCATCGAATAGA
GCTCTTTTTCGCTTAACTTGCTGCCGTTTTCCTCAGCATGCACAAGTTTGCTGATTAAATCG
TCTTTTGGCTTTATTCTTCTGTCATGGATCAGCTTAGCGATATAATCTTTAAATTCACGAAGG
GCCTGATTTGTCAGCTCTCTATTACCTTCAGAGGTATCAACCATCGCATTGGTCCAGATTTG
AAACTGTGACCGATCTTCTTTTGGGATTCCCATCAATTCAGATATAACAATAAAAGGCAAAG
GGGAAGCGAAGGATTTCATGATATCCGCTTTATTTTCTTTTTCCATTTCATCTAAAAGCTGTT
CAGCAATTTGTTCAATGCTGCCGCGCAGATTTTCAATGGTTCGGGGAGTAAATGCTTGATG
AACAAGTGATCTCAGGCGGGTATGGTCAGGTGTGTCTTTTGCCAGCATATGATCGGATACA
AAATCGATATCTTCACTAACGTTGAGCATTTTGATTTGTTCTTGGTTCATCACATTTTTTACG
TCTCTTGTAATTCGATTGTCTTTTAAAAAGGCCATACAATCATCGTATCGGGTAATTAACCAG
GCCGGATATGTGGCTCCGAACCGTTTTAATTCAAATCGGTGAATGGGCTCTTCCTCTCTAA
ATCGTCCTAAAACTGAAAAAGGATTGTGATGAAACTCTTTACCATGCGGATGAAACATCAAT

TTTTCCATTTGCATTCTCCTCGCCTAATAGGGTAAATAGATGAATCAAATTGCTGAATTAGTT
TACAAAAAACAGAATGATTTGAAATGTAATCCTGTCTCTAAAACTATATATCTATCTTAGGCG
TCATTCAATAGGGAGAAGAACGAAAAAAGTGAAAAAACGGCTCGATATAAAGCAGCGCCTT
TGAACGAAAGCTCAAAGGCGCTACGCTGTATTATTTTGATGAAAGTGGCTGTCAGCTGTGC
TGGATATCAATTGTATATACTGCACGATCTGTTACGACCTTCAATCCTTCGTTTTCTTGGTGA
ATATGAAGCTCACCTAATAAAGGAATCTCATAGGCATTTTGCGGAAGCGGTACGTTTCCGT
CCTCAGTGAATGTTGTTCCTTCGCCTTCTAAAACAAGCTCTTCTTTCGCAACATAATCATCA
GGATGATTCGTACTGCGTATAGCCACTTTTTGAAGGTGGAGAACAATCTGATCCAAGTCTG
ATATCTCTTCATCATGGGTCAATTCACCTTTTCTAATATCAAGCGTCTGGCCGACTTTTGATT
CCAGCCATTGAGAAAGCTGTGTATTCGTGTGTGCCATCAATATTCCTCCTTTTGATCTACAC
GATACTATTCCCAATTGCTGCATCTTTTACACGGGAAAGAGCCGCCCCACTCTTTATGTATG
AAACAGTTCGCTGTTGAGTGCCTTGATATAGCGTACGCATGCACGCGTTGATCAGGCGCCT
CGTTCCACTGAGCGTCAGACCCCGTAGAAAAGATCAAAGGATCTTCTTGAGATCCTTTTTTT
CTGCGCGTAATCTGCTGCTTGCAAACAAAAAAACCACCGCTACCAGCGGTGGTTTGTTTGC
CGGATCAAGAGCTACCAACTCTTTTTCCGAAGGTAACTGGCTTCAGCAGAGCGCAGATACC
AAATACTGTTCTTCTAGTGTAGCCGTAGTTAGGCCACCACTTCAAGAACTCTGTAGCACCG
CCTACATACCTCGCTCTGCTAATCCTGTTACCAGTGGCTGCTGCCAGTGGCGATAAGTCGT
GTCTTACCGGGTTGGACTCAAGACGATAGTTACCGGATAAGGCGCAGCGGTCGGGCTGAA
CGGGGGGTTCGTGCACACAGCCCAGCTTGGAGCGAACGACCTACACCGAACTGAGATAC
CTACAGCGTGAGCTATGAGAAAGCGCCACGCTTCCCGAAGGGAGAAAGGCGGACAGGTAT
CCGGTAAGCGGCAGGGTCGGAACAGGAGAGCGCACGAGGGAGCTTCCAGGGGGAAACG
CCTGGTATCTTTATAGTCCTGTCGGGTTTCGCCACCTCTGACTTGAGCGTCGATTTTTGTGA
TGCTCGTCAGGGGGGCGGAGCCTATGGAAAAACGCCAGCAACGCGGCCTTTTTACGGTTC
CTGGCCTTTTGCTGGCCTTTTGCTCACATGTTCTTTCCTGCGTTATCCCCTGATTCTGTGGA
TAACCGTATTACCGCCTTTGAGTGAGCTGATACCGCTCGCCGCAGCCGAACGACCGAGCG
CAGCGAGTCAGTGAGCGAGGAAGCGGAAGAGCGCCCAATACGCAAACCGCCTCTCCCCG
CGCGTTGGCCGATTCATTAATGCAGGTTGATCACCTAGAGGATCCCCCAAAGCATAAAAAC
TTGCATGGACTAATGCTTGAAACCCAGGACAATAACCTTATAGCTTGTAAATTCTATCATAAT
TGTGGTTTCAAAATCGGCTCCGTCGATACTATGTTATACGCCAACTTTGAAAACAACTTTGA
AAAAGCTGTTTTCTGGTATTTAAGGTTTTAGAATGCAAGGAACAGTGAATTGGAGTTCGTCT
TGTTATAATTAGCTTCTTGGGGTATCTTTAAATACTGTAGAAAAGAGGAAGGAAATAATAAAT
GGCTAAAATGAGAATATCACCGGAATTGAAAAAACTGATCGAAAAATACCGCTGCGTAAAA
GATACGGAAGGAATGTCTCCTGCTAAGGTATATAAGCTGGTGGGAGAAAATGAAAACCTAT
ATTTAAAAATGACGGACAGCCGGTATAAAGGGACCACCTATGATGTGGAACGGGAAAAGG
ACATGATGCTATGGCTGGAAGGAAAGCTGCCTGTTCCAAAGGTCCTGCACTTTGAACGGCA
TGATGGCTGGAGCAATCTGCTCATGAGTGAGGCCGATGGCGTCCTTTGCTCGGAAGAGTA
TGAAGATGAACAAAGCCCTGAAAAGATTATCGAGCTGTATGCGGAGTGCATCAGGCTCTTT
CACTCCATCGACATATCGGATTGTCCCTATACGAATAGCTTAGACAGCCGCTTAGCCGAAT
TGGATTACTTACTGAATAACGATCTGGCCGATGTGGATTGCGAAAACTGGGAAGAAGACAC
TCCATTTAAAGATCCGCGCGAGCTGTATGATTTTTTAAAGACGGAAAAGCCCGAAGAGGAA
CTTGTCTTTTCCCACGGCGACCTGGGAGACAGCAACATCTTTGTGAAAGATGGCAAAGTAA
GTGGCTTTATTGATCTTGGGAGAAGCGGCAGGGCGGACAAGTGGTATGACATTGCCTTCT
GCGTCCGGTCGATCAGGGAGGATATCGGGGAAGAACAGTATGTCGAGCTATTTTTTGACTT
ACTGGGGATCAAGCCTGATTGGGAGAAAATAAAATATTATATTTTACTGGATGAATTGTTTT
AGTACCTAGATTTAGATGTCTAAAAAGCCTGCAGTCGCGAGGCCTCGACGGATCCCCAAAG
CACTTGCATAGGCTAATGCCTGGCTTGGTTTGTTGAGACATTACCTGAGAGCCAAAAAACT
TTTTATGAATATGAATTAAAAAAAAGAACCAACAAAGGCTGAGACAGACTCCAAACGAGTCT
GTTTTTTTAAAAAAAATATTAGGAGCATTGAATATATATTAGAGAATTAAGAAAGACATGGGA

ATAAAAATATTTTAAATCCAGTAAAAATATGATAAGATTATTTCAGAATATGAAGAACTCTGTT
TGTTTTTGATGAAAAAACAAACAAAAAAAAATCCACCTAACGGAATCTCAATTTAACTAACAGC
GGCCAAACTGAGAAGTTAAATTTGAGAAGGGGAAAAGGCGGATTTATACTTGTATTTAACTA
TCTCCATTTTAACATTTTATTAAACCCCATACAAGTGAAAATCCTCTTTTACACTGTTCCTTTA
GGTGATCGCGGAGGGACATTATGAGTGAAGTAAACCTAAAAGGAAATACAGATGAATTAGT
GTATTATCGACAGCAAACCACTGGAAATAAAATCGCCAGGAAGAGAATCAAAAAAGGGAAA
GAAGAAGTTTATTATGTTGCTGAAACGGAAGAGAAGATATGGACAGAAGAGCAAATAAAAA
ACTTTTCTTTAGACAAATTTGGTACGCATATACCTTACATAGAAGGTCATTATACAATCTTAA
ATAATTACTTCTTTGATTTTTGGGGCTATTTTTTAGGTGCTGAAGGAATTGCGCTCTATGCTC
ACCTAACTCGTTATGCATACGGCAGCAAAGACTTTTGCTTTCCTAGTCTACAAACAATCGCT
AAAAAAATGGACAAGACTCCTGTTACAGTTAGAGGCTACTTGAAACTGCTTGAAAGGTACG
GTTTTATTTGGAAGGTAAACGTCCGTAATAAAACCAAGGATAACACAGAGGAATCCCCGATT
TTTAAGATTAGACGTAAGGTTCCTTTGCTTTCAGAAGAACTTTTAAATGGAAACCCTAATATT
GAAATTCCAGATGACGAGGAAGCACATGTAAAGAAGGCTTTAAAAAAGGAAAAAGAGGGTC
TTCCAAAGGTTTTGAAAAAAGAGCACGATGAATTTGTTAAAAAAATGATGGATGAGTCAGAA
ACAATTAATATTCCAGAGGCCTTACAATATGACACAATGTATGAAGATATACTCAGTAAAGG
AGAAATTCGAAAAGAAATCAAAAAACAAATACCTAATCCTACAACATCTTTTGAGAGTATATC
AATGACAACTGAAGAGGAAAAAGTCGACAGTACTTTAAAAAGCGAAATGCAAAATCGTGTC
TCTAAGCCTTCTTTTGATACCTGGTTTAAAAACACTAAGATCAAAATTGAAAATAAAAATTGT
TTATTACTTGTACCGAGTGAATTTGCATTTGAATGGATTAAGAAAAGATATTTAGAAACAATT
AAAACAGTCCTTGAAGAAGCTGGATATGTTTTCGAAAAAATCGAACTAAGAAAAGTGCAATA
AACTGCTGAAGTATTTCAGCAGTTTTTTTTATTTAGAAATAGTGAAAAAAATATAATCAGGGA
GGTATCAATATTTAATGAGTACTGATTTAAATTTATTTAGACTGGAATTAATAATTAACACGTA
GACTAATTAAAATTTAATGAGGGATAAAGAGGATACAAAAATATTAATTTCAATCCCTATTAA
ATTTTAACAAGGGGGGGATTAAAATTTAATTAGAGGTTTATCCACAAGAAAAGACCCTAATA
AAATTTTTACTAGGGTTATAACACTGATTAATTTCTTAATGGGGGAGGGATTAAAATTTAATG
ACAAAGAAAACAATCTTTTAAGAAAAGCTTTTAAAAGATAATAATAAAAAGAGCTTTGCGATT
AAGCAAAACTCTTTACTTTTTCATTGACATTATCAAATTCATCGATTTCAAATTGTTGTTGTAT
CATAAAGTTAATTCTGTTTTGCACAACCTTTTCAGGAATATAAAACACATCTGAGGCTTGTTT
TATAAACTCAGGGTCGCTAAAGTCAATGTAACGTAGCATATGATATGGTATAGCTTCCACCC
AAGTTAGCCTTTCTGCTTCTTCTGAATGTTTTTCATATACTTCCATGGGTATCTCTAAATGAT
TTTCCTCATGTAGCAAGGTATGAGCAAAAAGTTTATGGAATTGATAGTTCCTCTCTTTTTCTT
CAACTTTTTTATCTAAAACAAACACTTTAACATCTGAGTCAATGTAAGCATAAGATGTTTTTC
CAGTCATAATTTCAATCCCAAATCTTTTAGACAGAAATTCTGGACGTAAATCTTTTGGTGAAA
GAATTTTTTTATGTAGCAATATATCCGATACAGCACCTTCTAAAAGCGTTGGTGAATAGGGC
ATTTTACCTATCTCCTCTCATTTTGTGGAATAAAAATAGTCATATTCGTCCATCTACCTATCC
TATTATCGAACAGTTGAACTTTTTAATCAAGGATCAGTCCTTTTTTTCATTATTCTTAAACTGT
GCTCTTAACTTTAACAACTCGCAATCTTATCGGGCTATGCATGCTTACATCGTCCG

Oligonucleotides

Table S1: Oligonucleotides used in this study (underlined = overhang for homologous recombination, fw = forward primer, rv = reverse primer)

**Oligonucleotides**	**Sequence 5'-3'**	**Description**
14240	CGTCCATCTACCTATCCTATTATCG	Sequencing primer from low copy plasmid into left homology region (fw)
14159	GCAGCAGATTACGCGCAG	Sequencing primer from low copy plasmid into right homology region (rv)
02063	CGATACCGTTCAATCCATTCC	Sequencing primer from left homology region into insert (fw)
02065	GGCGTTTGTGTTTTCTCAG	Sequencing primer from right homology region into insert (rv)
02016	TACCGTTCGTATAGCATACATTATACGAAGTTATTAGACCGGTACTAAATTAAAGTAATAAAG	Amplification of spectinomycin resistance gene plus overhang containing *lox71* (fw)
02017	CTACCGTTCGTATAATGTATGCTATACGAAGTTATGGTCAGCTTTATTGAACAGTAATTTAAG	Amplification of spectinomycin resistance gene plus overhang containing *lox66* (rv)
14284	TTAACTTTAACAACTCGCAATCTTATCGGGCTATGCATGCACCTACAGCAAAGCAGGC	Amplification of 2 kb chromosomal fragment for 2x 1000 bp pksX homology region plus overhang to vector (fw)
14285	ACGCTCAGTGGAACGAGGCGCCTGATCAACGCGTGCATGCAAGACACACCTGACCATACC	Amplification of 2 kb chromosomal fragment for 2x 1000 bp pksX homology region plus overhang to vector (rv)
14286	TTAACTTTAACAACTCGCAATCTTATCGGGCTATGCATGCTCCCGCACCAATTAAGTAGG	Amplification of 1 kb chromosomal fragment for 2x 500 bp *pksX* homology region plus overhang to vector (fw)
14287	ACGCTCAGTGGAACGAGGCGCCTGATCAACGCGTGCATGCGCAGCCTGAAATGATCGC	Amplification of 1 kb chromosomal fragment for 2x 500 bp *pksX* homology region plus overhang to vector (rv)
14235	AGGCTAATGCCTGGCTTGGTTTGTTGAGACATTACCTGAGAGCC	Amplification of Bs ori from pHT01 plus overhang to Ec *ori* from pMSE3 (fw)

14236	CGGACGATGTAAGCATGCATAGCCCGATAAGATTGCGAGTTGTTAAAGTTAAGAGCAC	Amplification of Bs *ori* from pHT01 plus overhang to left 2 kb homology region (rv)
14153	CAATCTTATCGGGCTATGCATGCTTACATCGTCCGCTGCC	Amplification of 4 kb homology regions and Ec *ori* plus overhang to pHT01 (fw)
14238	GGCTCTCAGGTAATGTCTCAACAAACCAAGCCAGGCATTAG	Amplification of 4 kb homology regions and Ec ori plus overhang to pHT01 (rv)
14290	TAATGGTGAACCCTAAATAGAAGGAGGCGCACAAAATGAGTAAAGGAGAAGAACTTTTC	Amplification of *GFPmut2* from pYC121 plus overhang to P_{acoA} (fw)
14297	AATAACTTCGTATAATGTATGCTATACGAACGGTACTATTTGTATAGTTCATCCATGCC	Amplification of *GFPmut2* from pYC121 plus overhang to *lox71* (rv)
14167	CAGATCTTCCGGATGGCTCGAGTTTTTCAGCAAGATCATCCCTGCCAATAGTC	Amplification of 500 bp *pksX* left homology region plus overhang to pJET1.2 (fw)
14182	TTTGTGCGCCTCCTTCTATTTAG	Amplification of P_{acoA} (rv)
14298	ATAACTTCGTATAGCATACATTATACGAACGGTAGGATGAATTGGTGAAGCGCTG	Amplification of 500 bp *pksX* right homology region plus overhang to *lox66* (fw)
14181	AGCTGAGAATATTGTAGGAGATCTTCTAGAAAGATCTTGCTCGTTGTAGCCGG	Amplification of 500 bp *pksX* right homology region plus overhang to pJET1.2 (rv)
23043	ATCTTGCTGAAAAACTCGAGC	Amplification of pJET1.2 vector backbone (fw)
23044	ATCTTTCTAGAAGATCTCC	Amplification of pJET1.2 vector backbone (rv)
14326	GTCTCGTTTTACAGCATGCGGCCTCGAGGATCCAATTC	Amplification of left *pksX* homology region with overhang for P_{acoA} (rv)
14327	GAATTGGATCCTCGAGGCCGCATGCTGTAAAACGAGAC	Amplification of P_{acoA} with overhang for left *pksX* homology region (fw)
14332	AATAACTTCGTATAATGTATGCTATACGAACGGTAGGTACGTATCGCGAGAATTC	Amplification of *mPLC* gene with overhang to *lox71* (rv)
14333	AGAAGGAGGCGCACAAAATG	Sequencing primer for *mPLC* (fw)
